# Post-translational modifications of triosephosphate isomerase reveal metabolic vulnerabilities in T-ALL: effect of combining dichloroacetic acid and the PPI rabeprazole

**DOI:** 10.1042/BCJ20253451

**Published:** 2026-02-18

**Authors:** Yoalli Martínez-Pérez, Ignacio De la Mora-De la Mora, Gloria Hernández-Alcántara, Gabriela López-Herrera, Itzhel García-Torres, Saúl Gómez-Manzo, Alberto Olaya-Vargas, Gloria León-Avila, José Manuel Hernández-Hernandez, Fernando González-Rubio, C. Yusiel Flores-Braulio, Luis A. Flores-López, Sergio Enríquez-Flores

**Affiliations:** 1 Tecnológico de Monterrey, Escuela de Medicina y Ciencias de la Salud, Monterrey, Mexico City 14380, Mexico; 2 Laboratorio de Biomoléculas y Salud Infantil, Instituto Nacional de Pediatría, Mexico City 04530, Mexico; 3 Departamento de Bioquímica, Facultad de Medicina, Universidad Nacional Autónoma de México, Apartado Postal 70-159, Mexico City 04510, Mexico; 4 Laboratorio de Inmunodeficiencias, Instituto Nacional de Pediatría, Mexico City 04530, Mexico; 5 Laboratorio de Bioquímica Genética, Instituto Nacional de Pediatría, Mexico City 04530, Mexico; 6 Trasplante de Células Madre y Terapia Celular, Instituto Nacional de Pediatría, Mexico City 04530, Mexico; 7 Departamento de Zoología, Escuela Nacional de Ciencias Biológicas, Instituto Politécnico Nacional, Carpio y Plan de Ayala S/N, Casco de Santo Tomás, Mexico City 11340, Mexico; 8 Departamento de Biología Celular. CINVESTAV, Av. IPN 2508. Col., San Pedro Zacatenco, Mexico City 07360, Mexico; 9 Licenciatura en Biología, Facultad de Ciencias, Universidad Nacional Autónoma de México, Mexico City 04510, Mexico; 10 Laboratorio de Biomoléculas y Salud Infantil, SECIHTI-Instituto Nacional de Pediatría, Mexico City 04530, Mexico

**Keywords:** Metabolic reprogramming, post-translational modifications, Proton pump inhibitors (PPIs), T-cell acute lymphoblastic leukemia (T-ALL), Triosephosphate isomerase

## Abstract

Acute lymphoblastic leukemia, particularly the T-cell subtype, remains associated with poor outcomes in relapsed and adult patients, highlighting the need for novel therapeutic strategies. Metabolic reprogramming, especially glycolytic dependence, represents a promising target. Triosephosphate isomerase (TPI), a key glycolytic enzyme, undergoes cancer-associated post-translational modifications (PTMs), including deamidation and phosphorylation. Here, we evaluated the potential of proton pump inhibitors (PPIs), particularly rabeprazole (Rbz), to selectively target PTM-bearing TPI isoforms in Jurkat cell model. Recombinant TPI variants engineered to mimic PTMs exhibited increased reactivity toward thiol-modifying agents and higher predicted binding affinities for PPI compared with wild-type TPI. Consistent with these properties, biochemical assays demonstrated preferential inhibition of the deamidation- and phosphorylation-mimicking proteins, with Rbz significantly reducing their enzymatic activity. Native gel electrophoresis of Jurkat cells protein extracts revealed drug-induced accumulation of acidic TPI isoforms, whereas normal T lymphocytes predominantly retained unmodified TPI. Rbz selectively impaired intracellular TPI activity and viability in Jurkat cells, effects enhanced by dichloroacetate (DCA) co-treatment. This inhibition correlated with marked accumulation of methylglyoxal and advanced glycation end products. Finally, combined DCA–Rbz treatment induced extensive apoptotic death in Jurkat cells while sparing normal lymphocytes. These findings identify PTM-bearing TPI isoforms as selective metabolic vulnerabilities in Jurkat cells and support the potential repurposing of thiol-modifying agents, particularly, Rbz, as targeted antileukemic strategies.

## Introduction

Cancer remains one of the most pressing global health challenges, with leukemia contributing significantly to this burden. In 2022, leukemia ranked 13th among 36 major cancer types in terms of combined incidence and mortality, accounting for approximately 2.4% of all new cancer cases and 3.1% of cancer-related deaths worldwide [1].

Leukemia encompasses four major subtypes, among which acute lymphoblastic leukemia (ALL) is recognized as a genomically heterogeneous and biologically aggressive malignancy that affects individuals across all age groups. Notably, approximately 80% of ALL cases are diagnosed in pediatric patients, making it the most common form of childhood cancer [2]. Despite considerable therapeutic advances, ALL continues to be associated with substantial morbidity and, in specific patient subgroups, poor clinical outcomes [3].

ALL is further subclassified into B-cell and T-cell lineages based on the immunophenotype of malignant lymphoblasts, which originate from immature lymphoid precursors. B-cell ALL (B-ALL) constitutes approximately 75–80% of cases and predominates in pediatric populations. In contrast, T-cell ALL (T-ALL) accounts for 15–20% of cases [2] and is considered the more aggressive subtype, characterized by rapid disease progression, frequent relapse, and adverse clinical outcomes. These challenges are particularly pronounced in adult patients, where long-term remission is achieved in only about 35% of cases [2].

Although ALL is generally curable in approximately 80% of patients, the prognosis for those who relapse remains dismal, with survival rates dropping to around 20%. Despite significant progress in precision oncology, including the development of multi-agent chemotherapy regimens that overcome drug resistance and achieve high remission rates in relapsed ALL patients [4], treatment resistance and toxicity continue to limit the long-term efficacy of current therapeutic approaches. These enduring limitations underscore the critical need for innovative, more effective, and less toxic treatment strategies.

One promising area of exploration involves targeting metabolic reprogramming, a hallmark of cancer. Leukemic cells, like many other cancer types, undergo extensive metabolic remodeling to support uncontrolled proliferation, increased bioenergetic demands, and redox homeostasis. This metabolic shift favors anabolic growth and energy production, creating specific dependencies that can serve as therapeutic vulnerabilities.

Among the altered metabolic pathways, glycolysis plays a central role and is frequently up-regulated even in the presence of oxygen, a phenomenon known as the Warburg effect. A key enzyme in this pathway is triosephosphate isomerase (TPI), which catalyzes the reversible interconversion of dihydroxyacetone phosphate (DHAP) and glyceraldehyde-3-phosphate (G3P). This reaction ensures optimal utilization of glucose-derived intermediates, thereby maximizing ATP production and supporting biosynthetic processes.

Importantly, post-translational modifications (PTMs) of TPI, particularly deamidation at aminoacyl position 16 (N16D, dTPI) and phosphorylation at serine 21 (pTPI), have been identified in several cancer types, including T-ALL [5–7]. These modifications have been shown to alter enzymatic activity and structural stability, thereby influencing glycolytic flux and cancer cell metabolism [8]. Unlike the native isoform (wtTPI), dTPI and pTPI display reduced stability and distinct functional properties that may generate selective metabolic vulnerabilities. In particular, deamidation at N16 has been linked to the accumulation of isoforms in T-ALL, where selective inhibition of dTPI can impair leukemic cell viability without affecting normal T cells [6]. Similarly, phosphorylation at Ser21, mediated by LKB1-dependent kinases, modulates glycolytic flux in lung adenocarcinoma and other cancers, creating an additional regulatory layer with potential therapeutic relevance [5,8]. These vulnerabilities may be further accentuated if PTM-bearing isoforms are more abundantly expressed in malignant cells than in their normal counterparts, as has been demonstrated in T-ALL and other malignancies [6,7].

In this context, proton pump inhibitors (PPIs), initially developed to suppress gastric acid secretion, have recently garnered attention for their potential antitumor properties [9]. Rabeprazole (Rbz), a benzimidazole-based PPI, is of particular interest due to its demonstrated cytotoxic effects in cancer models through mechanisms independent of its classical pharmacological activity [10]. Additionally, dichloroacetate (DCA), a small molecule known to modulate mitochondrial metabolism, has shown promise in lowering the apoptotic threshold in leukemic cells, both alone and in combination with other metabolic inhibitors [11,12].

In this study, we propose that dTPI and pTPI isoforms represent a previously unrecognized, combined metabolic vulnerability in T-ALL that can be selectively targeted by PPIs, particularly Rbz. By evaluating the individual and combined effects of Rbz and DCA in Jurkat cells, a well-established T-ALL model, we aim to elucidate the functional contribution of these TPI isoforms to leukemic metabolism, with a focus on their role in methylglyoxal (MG) accumulation and the induction of apoptotic cell death.

## Results

### Functional and structural consequences of PTM-mimetic TPI variants reveal potential vulnerabilities to thiol-targeted compounds

To model PTMs, recombinant human TPI mutants were characterized. The N16D mutant (dTPI) mimics deamidation at Asn16, and the S21E mutant (pTPI) mimics phosphorylation at Ser21. Kinetic analyses revealed that each amino acid substitution had distinct functional consequences. The pTPI mutant displayed a 1.47-fold increase in *K*_M_ (1.13 mM vs 0.77 mM for wtTPI), accompanied by a modest 8% decrease in *V*_max_ (4639.6 vs 5044.4 μmol·min^−1^·mg^−1^). This resulted in a reduced turnover number (*k*_cat_ 4125 s^−1^ vs 4484 s^−1^ for wtTPI) and a 37% drop in catalytic efficiency (*k*_cat_/*K*_M_ = 3.65 × 10^6^ vs 5.82 × 10^6^ M^−1^·s^−1^) (Supplementary Figure S1 and Supplementary Table S1). In contrast, previous studies have shown that dTPI exhibits severe kinetic impairment, with a *k*_cat_/*K*_M_ reduced to 0.2 M^−1^·s^−1^ compared with 950 M^−1^·s^−1^ for wtTPI [13]. These kinetic profiles reveal distinct functional consequences of the two PTMs analyzed. Relative to the wtTPI, the pTPI retains substantial catalytic capacity despite reduced substrate affinity and catalytic efficiency, whereas the dTPI exhibits a notable impairment of catalytic efficiency. These differences suggest that phosphorylation and deamidation may function as independent and graded regulatory mechanisms in TPI activity.

**Figure 1:**
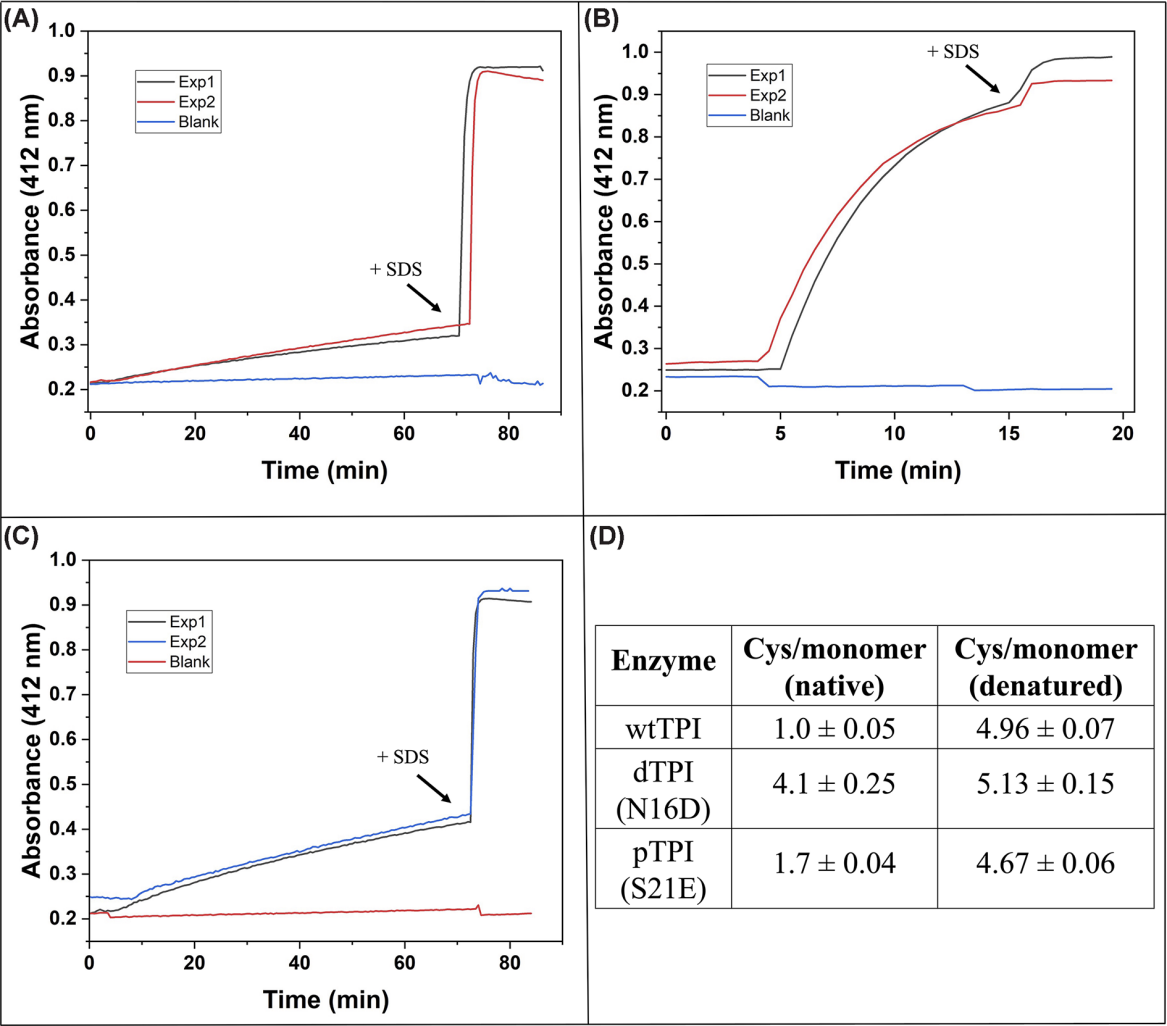
Cys accessibility in wtTPI, pTPI, and dTPI under native and denaturing conditions Thiol reactivity was evaluated by DTNB derivatization of recombinant TPI variants. Under native conditions, 200 μg of each enzyme was added, and TNB formation was monitored at 412 nm (**A–C**, black traces). After the incubation period, 5% SDS (arrows) was added to denature the proteins and expose all Cys residues fully. Red traces in panels (A) to (C) correspond to DTNB signals in TEA buffer with buffer aliquots added to match the dilution factor from protein addition. Panel (**D**) summarizes the number of titrated Cys residues.

Once the catalytic parameters were determined, the structural consequences of the PTMs were evaluated through thiol reactivity assays using 5,5′-dithiobis-(2-nitrobenzoic acid) (DTNB). Under native conditions, dTPI (N16D) showed the highest accessibility to cysteine (Cys), derivatizing near to 4 Cys per monomer within 15 min at 1 mM DTNB (Figure 1B,D). This represents a four-fold increase over wtTPI (near to 1.0 Cys/monomer) (Figure 1A,D) and is concordant with prior structural evidence that deamidation induces structural alteration near Cys residues [14]. The pTPI mutant exhibited intermediate reactivity (1.7 Cys/monomer after 70 min) (Figure 1C,D), suggesting that phosphorylation mimicry induces more localized or subtle structural perturbations compared with broader conformational changes in dTPI. Finally, to confirm the accessibility of DTNB to the five Cys residues per monomer present in the TPI amino acid sequence, SDS was added to each enzyme after the incubation period (Figure 1D).

Therefore, the increased sulfhydryl agent’s exposure of Cys in TPI with PTMs provides a structural rationale for selective drug targeting, as thiol-reactive compounds would preferentially bind to these forms.

### Molecular docking suggests that PTM-mimicking TPI mutants may display higher proton pump inhibitors binding affinity and interface-biased binding

Given the observed differences in DTNB reactivity between wtTPI and its PTM-mimicking variants, we next investigated their potential interactions with PPIs. PPIs are known to covalently modify Cys residues in target proteins [15], and their reactivity may be influenced by PTM-induced changes in thiol accessibility.

To investigate PPIs binding to different TPI variants, molecular docking analyses were performed using available crystallographic structures for wtTPI (PDB ID: 2JK2) [16] and deamidated mutant dTPI (PDB ID: 4UNK) [13]. The absence of a publicly available experimental structure for human TPI phosphorylated at Ser21 necessitated the generation of a 3D model for the pTPI (S21E) mutant. This strategy is well-supported, as crystallographic data for the analogous S20E mutant show its homodimer structure is virtually superimposable with wtTPI (RMSD: 0.25 Å), with the glutamate side chain adopting a solvent-exposed orientation that does not perturb the local fold [17]. Thus, to generate a 3D model of pTPI, was employed AlphaFold Colab v2 with dTPI structure as a template [18,19], followed by energy minimization to optimize geometry for docking.

**Figure 2:**
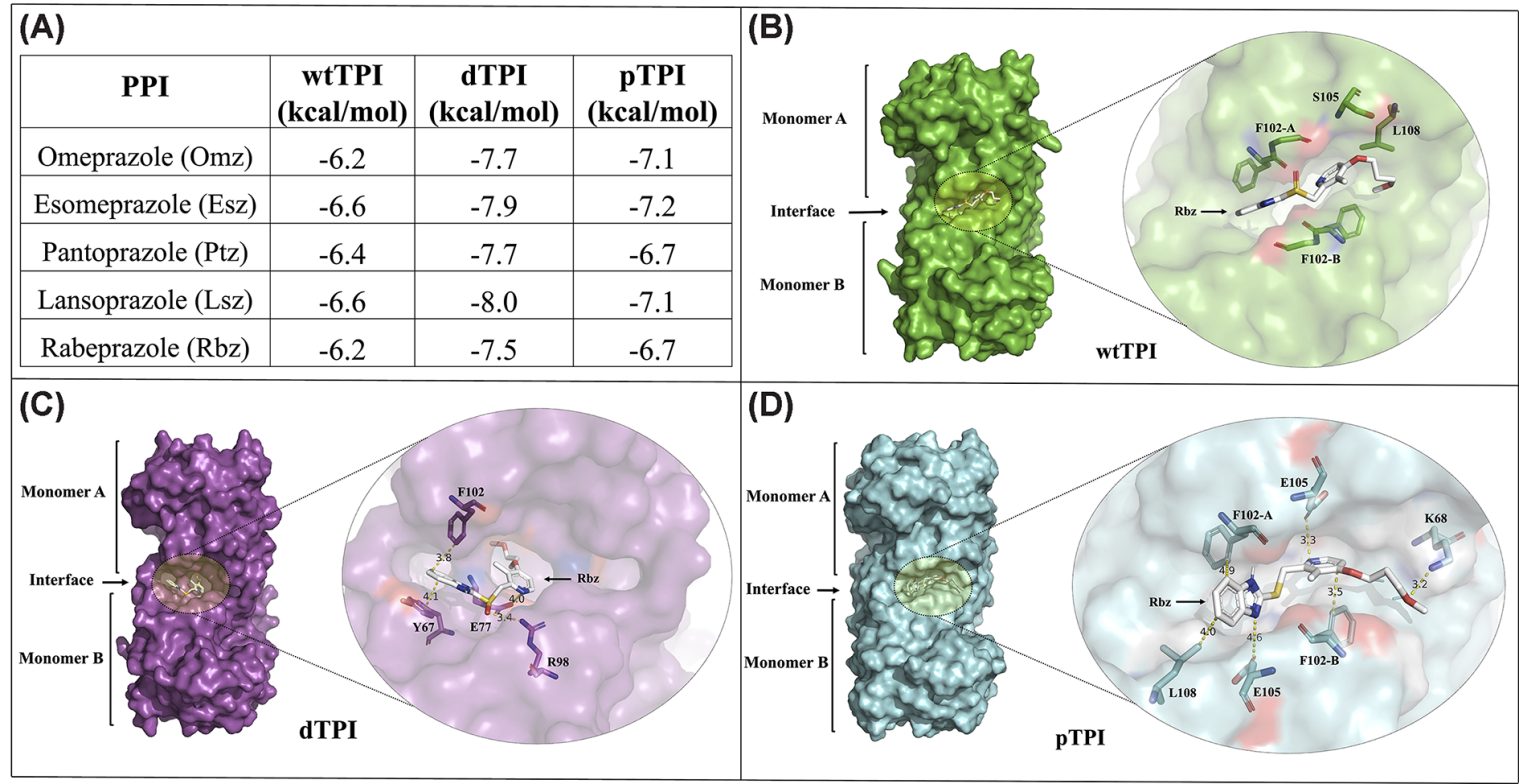
Molecular docking of PPIs on wtTPI and PTM-mimicking variants The 3D structures of wtTPI, dTPI, and pTPI were used for docking simulations with PPIs through the CB-Dock server as described in the Materials and methods section. (**A**) Predicted binding affinities of five PPIs for TPIs showed consistently stronger binding for PTM-mimicking variants. (**B**) Representative Rbz docking pose on wtTPI, showing limited surface-level contacts with Phe102 on both monomers. (**C**) Rbz binding to dTPI, highlighting π-stacking with Tyr67, hydrophobic interaction with Phe102, and polar contact between the sulfinyl oxygen (O3) and the NH2 of Arg98. (**D**) Rbz binding to pTPI, showing hydrophobic contacts with Leu108 and Phe102 and a potential electrostatic interaction between the benzimidazole nitrogen and Glu105. All docking poses were visualized with PyMOL Molecular Graphics System v2.5.0 (Schrödinger, LLC, New York, NY, U.S.A.).

Molecular docking revealed PTM-dependent differences in PPIs binding to TPI (Figure 2A). Relative to wtTPI, which showed binding energies between −6.2 and −6.6 kcal/mol, and predominantly interface-localized poses, with pantoprazole (Ptz) favoring the catalytic pocket, the PTM-mimicking variants exhibited consistently stronger predicted binding and a dominant preference for the dimer interface. Across all PPIs and TPI variants, the interface cavity corresponded to the best-ranked binding mode, with only a single exception in which it represented the second-best pose. Binding energies improved by approximately 1.1–1.4 kcal/mol in dTPI and by ∼0.5–0.6 kcal/mol in pTPI relative to wtTPI. Among the PTM mimics, lansoprazole (Lsz) emerged as the most effective binder in dTPI, whereas esomeprazole (Esz) showed the strongest predicted interaction in pTPI. The five highest-ranked binding poses for each PPI–TPI combination are reported in the Supplementary Material (Supplementary Table S2).

Detailed analysis of Rbz docking poses, selected as a representative PPI, provided further mechanistic insight into these differences (Figure 2B–D). In wtTPI, Rbz formed only superficial contacts, principally involving Phe102 for both monomers (Figure 2B). In contrast, docking to dTPI revealed a more extensive interaction network, including π-stacking interaction between the benzimidazole ring of Rbz and Tyr67 (4.1 Å), a hydrophobic interaction with Phe102 (3.2 Å), and a polar contact between the sulfinyl group (O3) of Rbz and the NH_2_ group of Arg98 (Figure 2C). Similarly, in pTPI, Rbz engaged in hydrophobic interactions with Leu108 and Phe102, and showed a potential electrostatic interaction between the nitrogen atom of the benzimidazole ring and the OE1 of Glu105 (Figure 2D). Additional hydrophobic and electrostatic contacts were observed in the pyrimidyl region of Rbz, indicating an expanded binding surface and improved complementarity at the dimer interface of the PTM-mimicking variants.

Together, these findings indicate that the computational docking analyses are consistent with and reinforce the biochemical data (Figure 1), linking the experimentally observed increase in Cys accessibility with enhanced PPI binding; although these interactions represent probabilistic predictions based on static structural models and selected ligands, particularly in the absence of an experimentally resolved pTPI structure, the convergence of docking trends across multiple PPIs, when interpreted alongside DTNB derivatization data, supports the robustness and biological relevance of the proposed binding mechanisms in TPI and its post-translationally modified proteoforms.

Given this differential interaction profile, the next experiment was used to experimentally validate whether PPIs exhibit selective targeting of PTM-modified TPI variants.

### PPIs selectively inhibit dTPI and pTPI phosphomimetic variants

**Figure 3:**
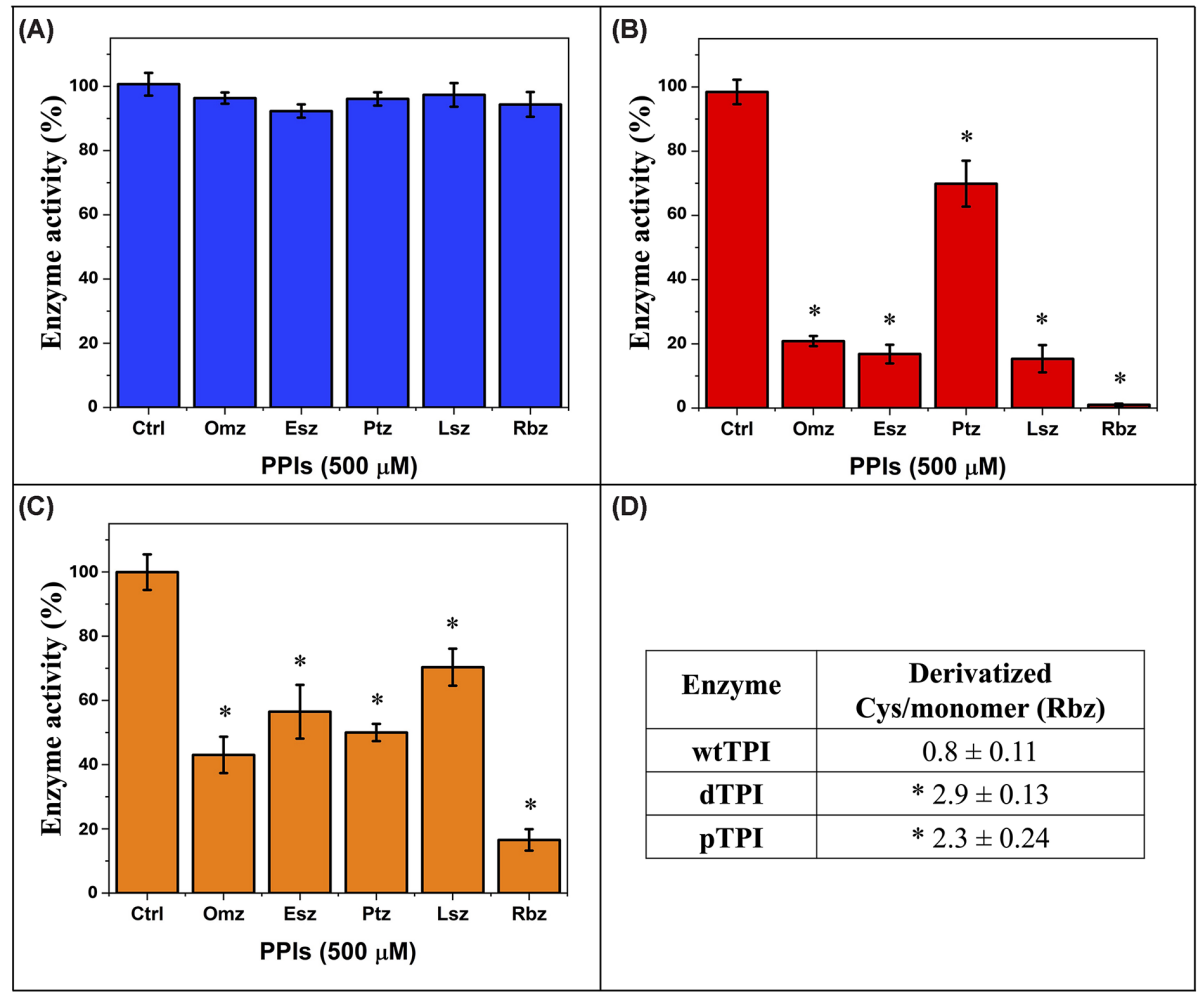
Enzymatic activity of TPI variants following PPI treatment wtTPI, dTPI, and pTPI were incubated for 2 h either under control conditions (Ctrl) or in the presence of 500 μM of the indicated PPIs. Residual enzymatic activity was subsequently measured as described in the Materials and methods section. (**A**) wtTPI exhibited negligible inhibition with all PPIs. (**B**) dTPI activity was completely abolished by Rbz, with moderate inhibition by Ptz and minimal effects from Omz, Esz, and Lsz. (**C**) pTPI showed strong inhibition by Rbz, moderate inhibition by Omz and Ptz, and minimal effects with Esz and Lsz. (**D**) Table of derivatized Cys residues/monomer following Rbz treatment, indicating the highest modification of Cys in dTPI, followed by pTPI and wtTPI. Data represent the mean ± SD of three independent biological replicates. Statistical significance was determined via one-way ANOVA followed by Tukey’s post-hoc test *P*<0.05 (*). To allow visualization of the variability and distribution of the underlying data, all individual biological replicate values are provided in the Supplementary Material (Supplementary Figure S2).

To evaluate the effect of PPIs on TPI variants, recombinant wtTPI, dTPI (N16D), and pTPI (S21E) were incubated with 500 μM of five different PPIs. Residual enzymatic activity measurements revealed marked selectivity for the modified TPI variants, with scarce effects on wtTPI (residual activities above 91%; Figure 3A). Statistical analysis using one-way ANOVA followed by Tukey’s post-hoc test confirmed that all PPIs significantly inhibited the enzymatic activity of both dTPI and pTPI compared with the control (*P*<0.05). Among the compounds tested, Rbz was the most effective inhibitor, virtually abolishing dTPI activity (Figure 3B) and reducing pTPI activity to 17% (Figure 3C). This was followed by omeprazole (Omz) and Esz, while Ptz and Lsz exhibited the least inhibitory effect. Notably, dTPI remained consistently more susceptible to inhibition than pTPI across all treatments, aligning with previous reports regarding dTPI sensitivity to Rbz [20].

To explore the molecular basis of this selectivity, was quantified the number of derivatized Cys residues per TPI monomer following Rbz treatment. dTPI exhibited the highest level of derivatization (approaching 3 Cys/monomer), followed by pTPI (∼2 Cys/monomer) and wtTPI (∼1 Cys/monomer) (Figure 3D). Statistical analysis confirmed that both dTPI and pTPI were significantly more sensitive to the chemical modification of Cys residues than wtTPI (*P*<0.05). The strong correlation between Cys accessibility and inhibition profiles suggests that PTMs structurally reposition Cys residues, thereby enhancing their reactivity with PPIs. Given that these TPI modifications are documented in T-ALL [6], the selective inhibition of these variants at clinically relevant concentrations (150–500 μM) strengthens the potential of repurposing PPIs as targeted therapies. Furthermore, the agreement between our computational models and biochemical data validates the use of PTM-mimicking mutants (N16D and S21E) as tools for identifying selective inhibitors of enzymatic proteoforms that may occur in cancer cells.

### Rabeprazole induces thermal destabilization in PTM-mimicking TPI variants

Thermal denaturation assays using Far-UV Circular Dichroism (CD) spectroscopy monitored at 222 nm were performed to characterize the structural consequences of Rbz interaction and the effect of modifications on protein stability.

**Figure 4:**
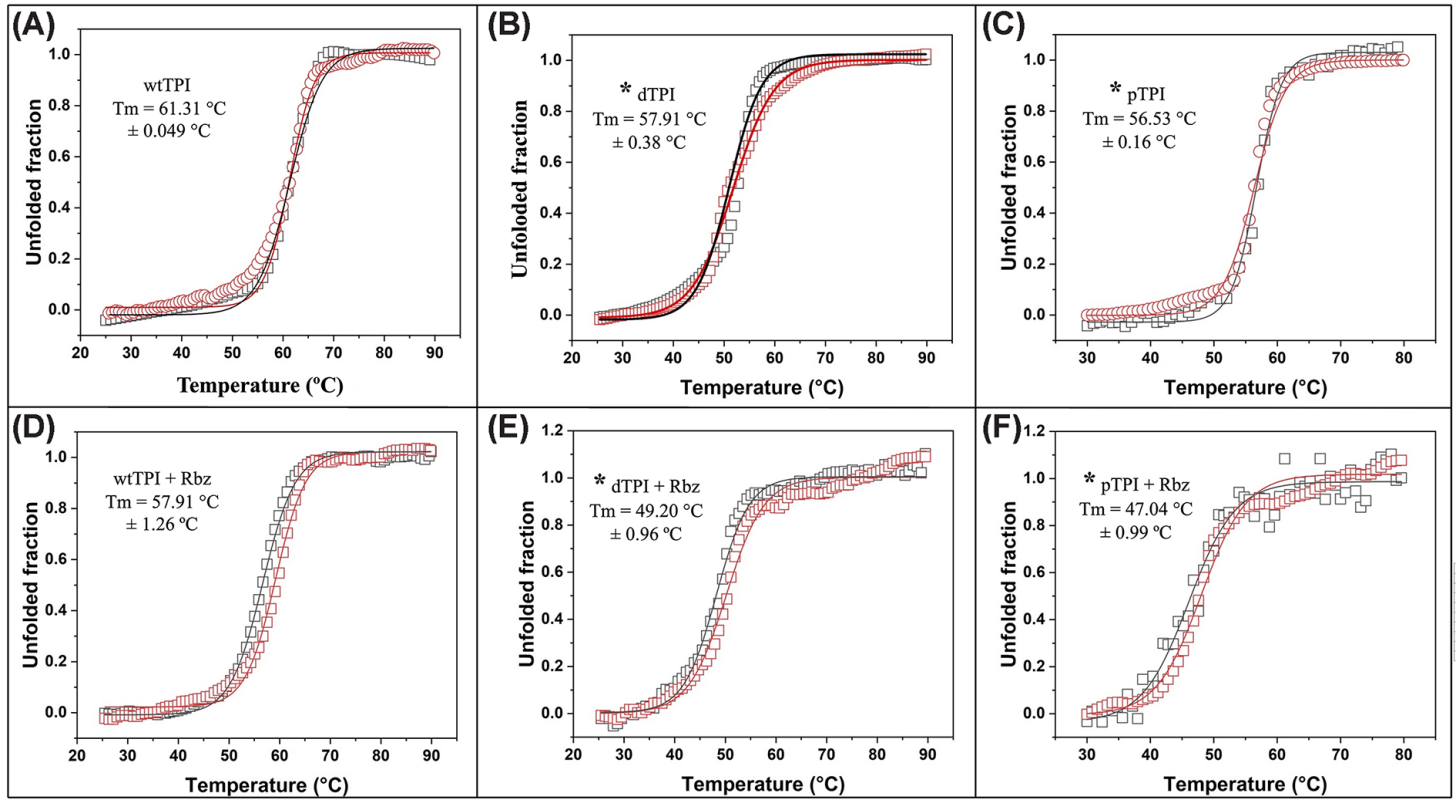
Thermal denaturation of wtTPI, dTPI, and pTPI with and without Rbz Proteins (0.2 mg/ml) were unfolded by increasing the temperature from 25–30°C to 80–90°C at a rate of 1°C per 2.5 min, with monitoring via CD at 222 nm. (**A**–**C**) without treatment; (**D–F**) treatment with Rbz. Data points represent the fraction of unfolded protein, and lines indicate Boltzmann fits used to calculate *T*_m_ values. *T*_m_ values represent the mean ± SD of two independent biological experiments. Statistical analysis was performed using one-way ANOVA followed by Tukey’s post-hoc test; statistical significance was defined as *P*<0.05 (*).

The results demonstrated that dTPI and pTPI significantly compromise the global conformational stability of their scaffold. While wtTPI exhibited a melting temperature (*T*_m_) of 61.31°C, dTPI and pTPI showed significantly lower values of 57.91°C and 56.53°C, respectively (Figure 4A–C). These shifts represent a substantial decrease in thermal stability of approximately 3°C and 5°C compared with the WT enzyme, with one-way ANOVA confirming these reductions as statistically significant (*P*<0.05).

We further evaluated the synergistic effect of Rbz on the *T*_m_ of all TPIs. Incubation with the inhibitor induced an additional *T*_m_ decrease in all enzymes. In the case of wtTPI, the *T*_m_ decreased to 57.91°C, indicating limited structural perturbation. Conversely, dTPI and pTPI exhibited more pronounced thermal destabilization, with *T*_m_ values dropping sharply to 49.20°C and 47.04°C, respectively (Figure 4D–F). Statistical analysis confirmed that the Rbz-induced decrease in *T*_m_ for both mutants was significantly greater than that observed for the WT (*P*<0.05). These data indicate a general reduction of approximately 8°C in the *T*_m_ of both modified variants relative to the WT under inhibitory conditions. This reinforces the premise that PTM-mimicking variants are not only intrinsically less stable but also inherently more susceptible to compound-induced structural destabilization. The pattern of thermal denaturation observed here correlates strongly with the previously described enzymatic inactivation profiles (Figure 3), supporting the hypothesis that PPIs selectively impair modified TPI variants by targeting conformationally vulnerable or destabilized proteoforms.

### Pharmacological treatment induces accumulation of modified TPI isoforms in Jurkat cells

In a previous study, we established that dTPI accumulates in Jurkat cells under drug treatment [6]. To comprehensively characterize the PTMs status of TPI in normal T lymphocytes and Jurkat cells, an electrophoretic analysis was performed using charge-sensitive native PAGE (nPAGE). This technique effectively separates protein isoforms based on their charge-to-mass ratio, enabling resolution of differentially modified TPI variants.

**Figure 5:**
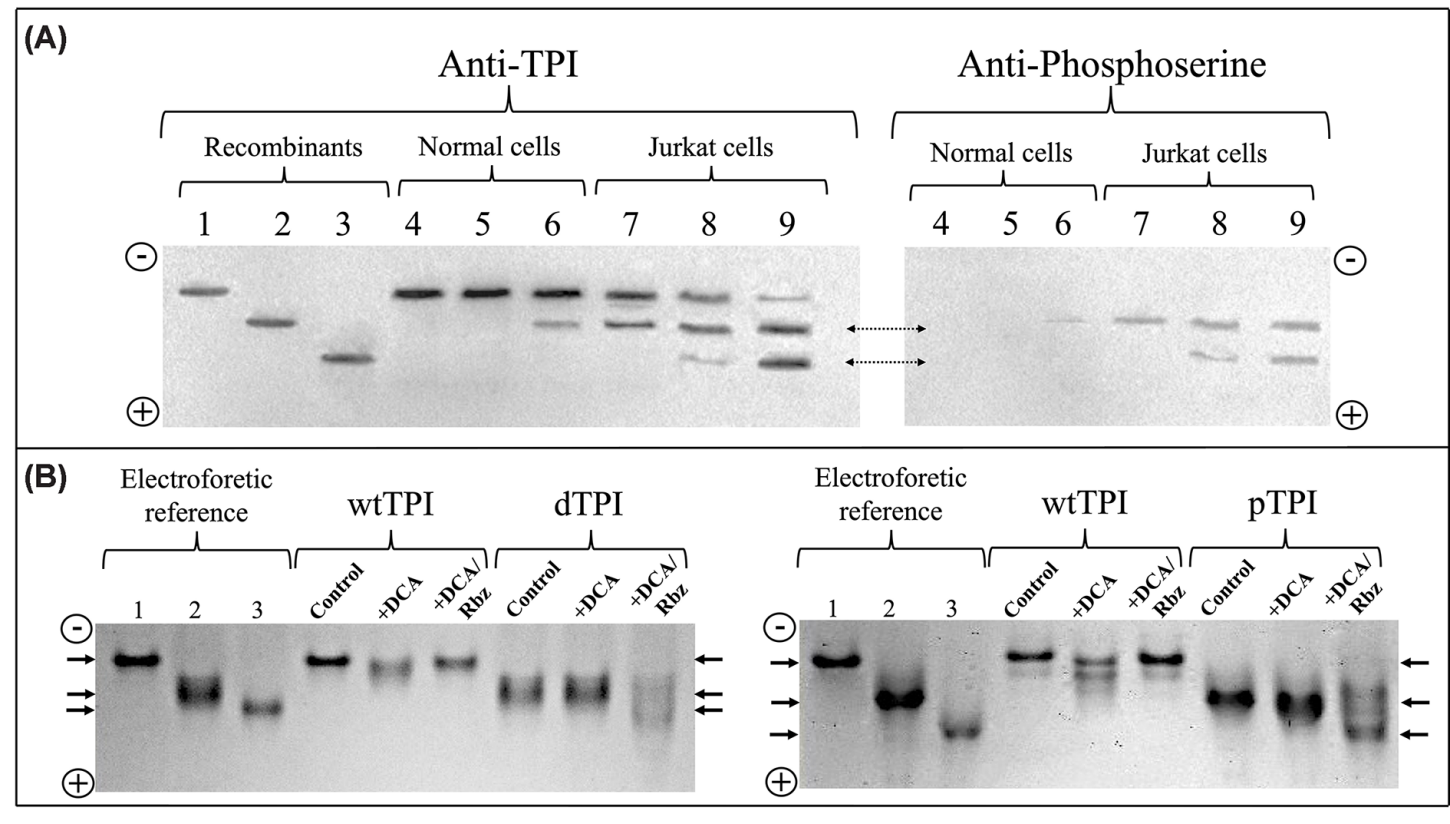
nPAGE analysis of TPI isoforms in normal T lymphocytes, Jurkat cells, and recombinant TPIs under drug treatment (**A**) Left blot: Anti-TPI immunoblot. Lanes 1–3 contain 1 μg of purified recombinant wtTPI, monodeamidated dTPI (N16D), and doubly deamidated ddTPI (N16D/N72D), used as migration standards. Lanes 4–6 contain immunoprecipitated TPI from normal T lymphocytes; lanes 7–9 contain immunoprecipitated TPI. Right blot: Anti-phosphoserine immunoblot of nPAGE-resolved, immunoprecipitated TPI from the same samples. The upper dashed arrow marks phospho-positive bands migrating at the dTPI position; the lower dashed arrow marks phospho-positive bands at the ddTPI position. Densitometric analysis of the bands is shown in Supplementary Figure S3. (**B**) Coomassie-stained nPAGE. Lanes 1–3 (left and right gels) contain TPI migration standards. In the left gel, lanes 4 and 5 contain wtTPI incubated with DCA alone or DCA–Rbz, respectively; lanes 6 and 7 contain dTPI under the same treatments. In the right gel, lanes 4 and 5 contain wtTPI treated with DCA or DCA–Rbz; lanes 6 and 7 contain pTPI treated identically. Drug-induced shifts in mobility toward more acidic isoforms are indicated, with ddTPI positions marked for reference. All gels were run under native conditions. Full-length, uncropped blots and gels for panels (A) and (B) are provided in Supplementary Figures S4–S7.

Using purified recombinant standards, we first established reference migration patterns for wtTPI, mono-deamidated dTPI (N16D), and doubly-deamidated ddTPI (N16D/N72D). As expected, these isoforms displayed progressively faster anodal migration corresponding to their increasing negative charge. Specifically, wtTPI showed the slowest mobility, dTPI migrated further, and ddTPI showed the highest mobility toward the anode (Figure 5A, lanes 1–3).

In normal T lymphocytes, a single TPI band was detected, comigrating precisely with wtTPI in both untreated cells and those treated with DCA alone (Figure 5A, lanes 4 and 5). While combined DCA–Rbz treatment (12 mM DCA + 500 μM Rbz), induced the appearance of a more acidic isoform (9% of total TPI by densitometry, Supplementary Figure S3) migrating at the dTPI position (Figure 5A, lane 6). Since DCA is a known inhibitor of pyruvate dehydrogenase kinase (PDK) and is used to modulate tumor metabolism, its lack of effect on TPI migration in normal T cells suggests that this intervention does not trigger significant PTMs such as deamidation or phosphorylation under these conditions.

Jurkat cells, however, exhibited a more complex isoform pattern that was highly responsive to drug treatment (Figure 5A, lanes 7–9). Untreated cells displayed two main bands: a predominant species comigrating with wtTPI (68.9% of total TPI) and a secondary band at the dTPI position (20.4%) (Figure 5A, lane 7). DCA treatment increased the acidic dTPI-like species to 30.7% and induced an additional band migrating at the ddTPI position (10.3%) (Figure 5A, lane 8, and Supplementary Figure S3). The DCA–Rbz combination produced even more pronounced changes, reducing the wtTPI band to 6.8% and enriching acidic isoforms, dTPI (37.7%) and ddTPI (41%), compared with the TPI profile of normal T lymphocytes (Figure 5A, lane 4) with 100% of the band migrating at the wtTPI position.

To assess phosphorylation status, the same membrane used for anti-TPI detection was probed with anti-phosphoserine antibodies following TPI immunoprecipitation (Figure 5A, right blot). In normal T lymphocytes, TPI phosphorylation was undetectable under control or DCA treatment (lanes 4 and 5, respectively), but DCA–Rbz exposure yielded a weak phospho-positive band at the dTPI position (2.7% by densitometry) (lane 6). In Jurkat cells, basal TPI phosphorylation was already evident (8.1%), appearing as a phospho-positive band at the dTPI position (Figure 5A, lane 7, upper dashed arrow). DCA treatment increased this signal to 16.2% and induced a second, more acidic phospho-band at the ddTPI position (8.1%), indicating enhanced TPI phosphorylation (lane 8). Combined DCA–Rbz treatment further enhanced these modifications, producing strong phospho-signals at both dTPI (21.3%) and ddTPI (23.4%) positions (lane 9, upper and lower dashed double arrows).

Overall, these results demonstrate that drug exposure promotes the accumulation of multiple acidic TPI isoforms in leukemic cells, whereas normal T cells largely retain unmodified TPI. The drug-induced accumulation of these acidic variants, particularly the appearance of doubly modified TPI following DCA–Rbz treatment, is consistent with results from recombinant PTM-mimicking mutants (Figure 3). Furthermore, the observed TPI deamidation and phosphorylation suggest a potential mechanism underlying its induction and accumulation following drug treatment.

To further dissect the contribution of DCA and Rbz to TPI modification, we performed nPAGE using recombinant TPIs (wtTPI, dTPI, and pTPI), treated with DCA or the DCA–Rbz combination (Figure 5B). Lanes 1–3 of both left and right gels show reference migration patterns for wtTPI, dTPI, and ddTPI, respectively, serving as molecular landmarks for detecting acidic species.

In the left gel of Figure 5B, wtTPI remained unaltered in mobility upon incubation with DCA alone or DCA–Rbz, suggesting that these treatments do not promote spontaneous deamidation under cell-free conditions. Similarly, dTPI did not yield additional acidic species when incubated with DCA alone. However, in the presence of the DCA–Rbz combination, a relatively diffuse band appeared at the ddTPI position, suggesting an additive or synergistic effect of the drugs in promoting further acidic modification.

As observed in the right gel of Figure 5B, incubation of wtTPI with DCA led to the emergence of more acidic isoforms, implying a drug-induced change in charge state, possibly via an indirect mechanism or a subtle conformational shift that facilitates the induction of negative charges (possibly deamidation). Intriguingly, this effect was not observed when wtTPI was treated with both DCA and Rbz, suggesting that Rbz may counteract or inhibit the modification pathway triggered by DCA. In contrast, pTPI did not exhibit increased acidic forms upon DCA treatment alone, but the DCA–Rbz combination induced a marked shift toward the ddTPI position, consistent with the accumulation of doubly acidic species.

These results reinforce the cell-based findings by demonstrating that DCA, particularly in combination with Rbz, promotes or stabilizes the generation of acidic TPI isoforms. While normal T lymphocytes resist these modifications even under drug pressure, leukemic Jurkat cells exhibit a distinctive vulnerability to metabolic modulation, resulting in the accumulation of modified TPI variants. These observations not only highlight the specificity of TPI post-translational remodeling in malignant cells but also strongly suggest that DCA–Rbz treatment may selectively exacerbate or reveal metabolic liabilities exploitable for therapeutic intervention.

### Selective cytotoxicity of rabeprazole in leukemic cells correlates with TPI inhibition

After identifying modified TPI isoforms in Jurkat cells, we assessed whether these alterations confer differential sensitivity to Rbz in normal versus leukemic lymphocytes.

**Figure 6:**
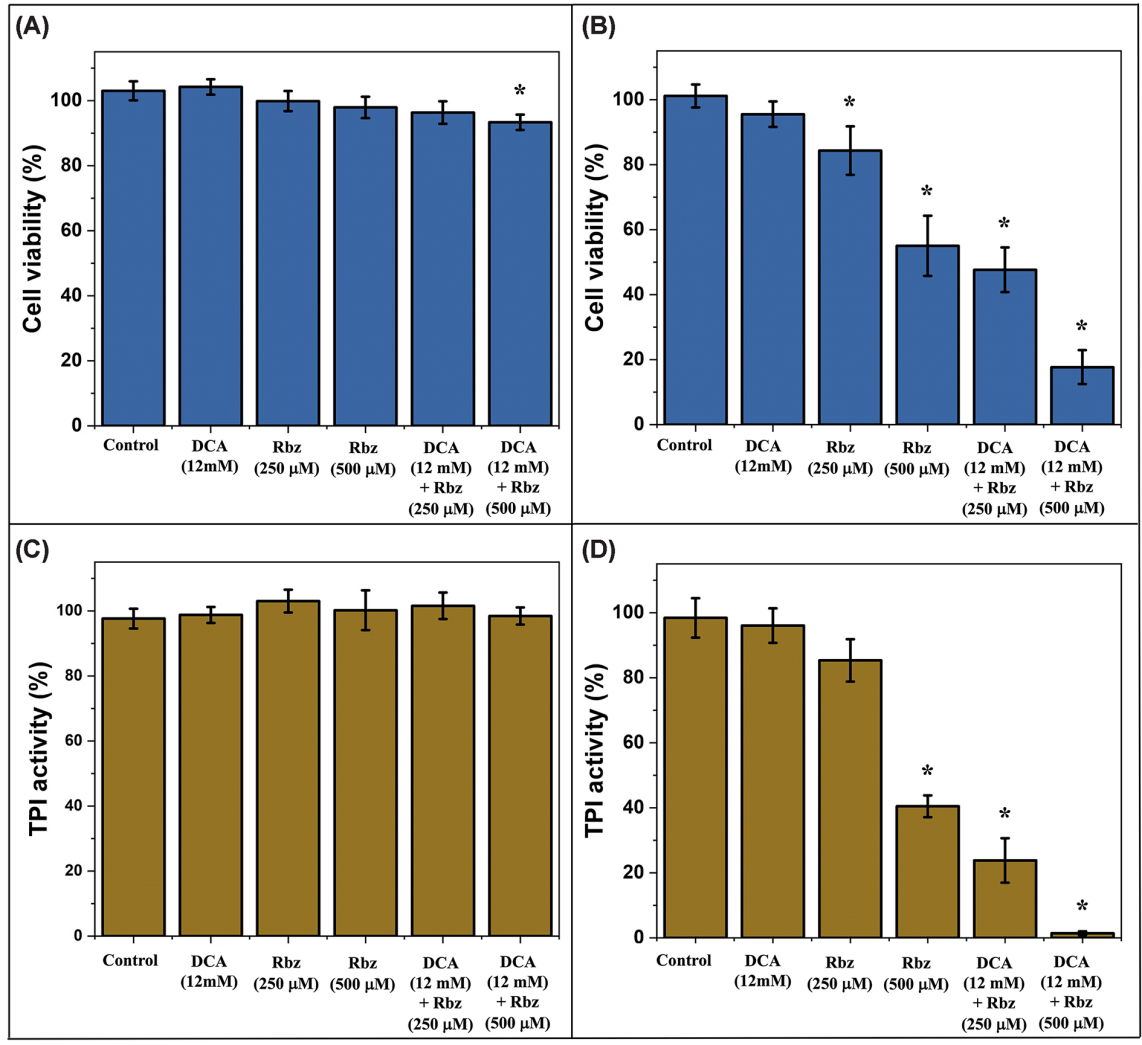
Effects of DCA and Rbz on cell viability and and endogenous TPI activity Normal and Jurkat T cells (1 × 10^5^ cells/well) were treated for 24 h with 12 mM DCA, Rbz (250 or 500 μM), or their combinations. (**A,B**) Correspond to cell viability measured by MTT assay. (**C,D**) Correspond to endogenous TPI activity determined spectrophotometrically. (A,C) Represent Normal T lymphocytes. (B,D) Represent Jurkat T cells. Results are expressed as percentages relative to untreated controls (set to 100%) and represent three independent biological replicates (mean ± SD). Statistical analysis was performed using one-way ANOVA followed by Tukey’s post-hoc test; statistical significance was defined as *P*<0.05 (*). To allow visualization of the variability and distribution of the underlying data, all individual biological replicate values are provided in the Supplementary Material (Supplementary Figure S8).

Normal T lymphocytes demonstrated robust resistance to all treatments, exhibiting less than 10% loss of viability after 24 h exposure to 12 mM DCA, 250 μM Rbz, 500 μM Rbz or their combination (Figure 6A). Statistical analysis (one-way ANOVA with post-hoc Tukey) confirmed that normal lymphocytes remained practically unaltered across all conditions (*P*>0.05). Consistent with this trend, Jurkat cells showed a minimal, non-significant response to DCA monotherapy (approximately 5% loss of viability). Strikingly, however, Rbz monotherapy induced significant, concentration-dependent cytotoxicity in Jurkat cells, with 20% and 40% viability losses at 250 and 500 μM, respectively (*P*<0.05), revealing a leukemic-specific vulnerability (Figure 6A,B).

This selectivity was further amplified in combination therapy: while normal T lymphocytes maintained 94% viability under 12 mM DCA + 250 or 500 μM Rbz co-treatment, Jurkat cells suffered synergistic cell death (53% and 80% loss of viability, respectively), which represented a statistically significant reduction compared with control groups (*P*<0.05) (Figure 6B). The potentiation of Rbz cytotoxicity by DCA is concordant with the DCA–Rbz-mediated enrichment of modified TPI isoforms in Jurkat cells (Figure 5A), suggesting these isoforms may sensitize leukemic cells to drug action.

Parallel measurements of intracellular TPI activity demonstrated mechanistic consistency with the cell viability results. Normal T lymphocytes maintained 92% of baseline activity under all treatment conditions (Figure 6C), with no significant enzymatic impairment observed. In contrast, Jurkat cells exhibited significant reductions in TPI activity; while 250 μM Rbz and 12 mM DCA alone showed minimal impact, treatment with 500 μM Rbz induced a significant 60% reduction in activity (*P*<0.05) (Figure 6D). Furthermore, co-administration with DCA significantly inhibited TPI function, with activity dropping by 80% and 99% at 250 and 500 μM Rbz, respectively (*P*<0.05) (Figure 6D). The close correlation between viability loss and TPI inhibition supports an on-target mechanism through TPI targeting.

### Acute rabeprazole exposure selectively inhibits TPI activity in leukemic cells

**Figure 7:**
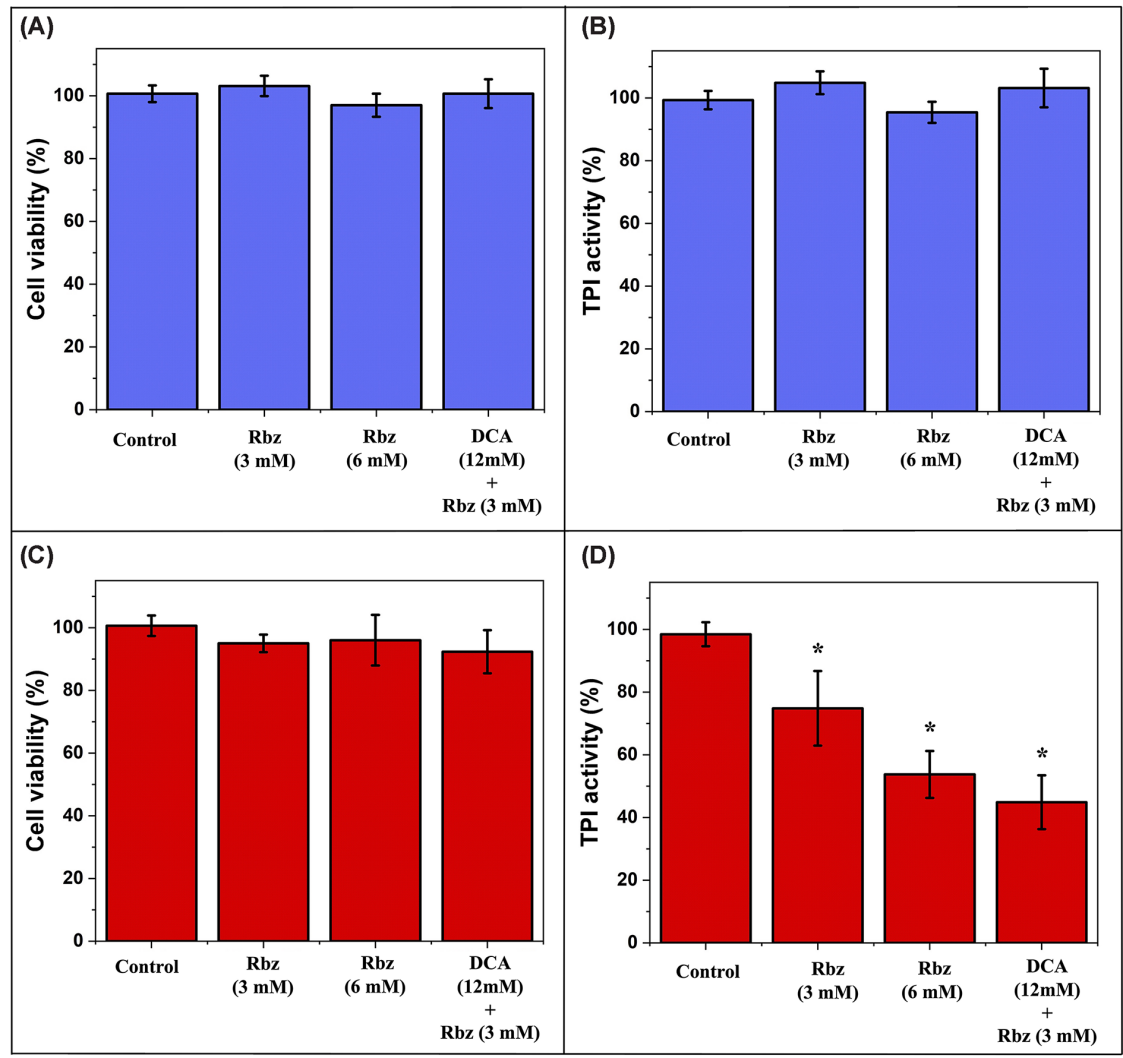
Evaluation of acute Rbz exposure in normal T lymphocytes and Jurkat cells Normal T lymphocytes (**A**,**B**) and Jurkat cells (**C**,**D**) (1 × 10^5^ cells per well) were exposed for 3 h under standard culture conditions to Rbz alone (3 or 6 mM) or to 12 mM DCA followed by 3 mM Rbz. Cell viability was assessed using the MTT assay (**A,C**), and intracellular TPI activity was determined spectrophotometrically in clarified cell lysates (**B**,**D**). Data are expressed as percentages relative to untreated controls (set to 100%) and represent the mean ± SD of three independent biological replicates. Statistical analysis was performed using one-way ANOVA followed by Tukey’s post-hoc test; statistical significance was defined as *P*<0.05 (*). To visualize data variability and distribution, all individual biological replicate values are shown in Supplementary Figure S10.

To distinguish direct enzymatic inhibition from secondary cytotoxic effects, we performed acute exposure experiments with Rbz under conditions that preserved cell viability (i.e., viability greater than 90% after 3 h of drug exposure). When viability was assessed in both normal T lymphocytes and Jurkat cells treated with 3 or 6 mM Rbz alone, as well as 12 mM DCA plus 3 mM Rbz, it consistently remained above 88% in all conditions (Figure 7A,C, respectively), confirming that any observed biochemical alterations were not attributable to overt cytotoxicity. Despite this preserved viability, intracellular TPI activity was differentially affected. In normal T lymphocytes, activity remained near 100% under all conditions with no significant deviations (Figure 7B, *P*>0.05). In contrast, Jurkat cells exhibited a significant, progressive decline in intracellular TPI activity. Statistical analysis using one-way ANOVA followed by Tukey’s post-hoc test demonstrated that the reductions of approximately 23%, 44%, and 58% observed upon treatment with 3 and 6 mM Rbz and the DCA–Rbz combination, respectively, were all statistically significant (*P*<0.05, Figure 7D).

To further characterize the effect of Rbz on TPI activity in leukemic cells, was performed kinetic analyses using lysates from Jurkat cells treated with 6 mM Rbz, a concentration sufficient to saturate the target without affecting cell viability. The substrate affinity was only slightly altered, with the Michaelis constant (*K*_M_) increasing modestly from 0.58 mM in controls to 0.76 mM following Rbz treatment, indicating largely preserved substrate binding. In contrast, the maximum velocity (*V*_max_) decreased substantially from 9.98 to 5.4 μmol·min^−1^·mg^−1^, corresponding to an approximate 46% reduction in catalytic capacity (Supplementary Figure S9 and Supplementary Table S3).

These findings demonstrate that Rbz induces significant inhibition of TPI catalytic activity in Jurkat cells, occurring prior to any detectable cytotoxicity. Together, the data provide compelling evidence that Rbz exerts a direct and selective inhibitory effect on TPI enzymatic function in leukemic cells, independent of cell death.

### Differential accumulation of methylglyoxal and advanced glycation end products is associated with TPI inhibition in Jurkat cells

**Figure 8:**
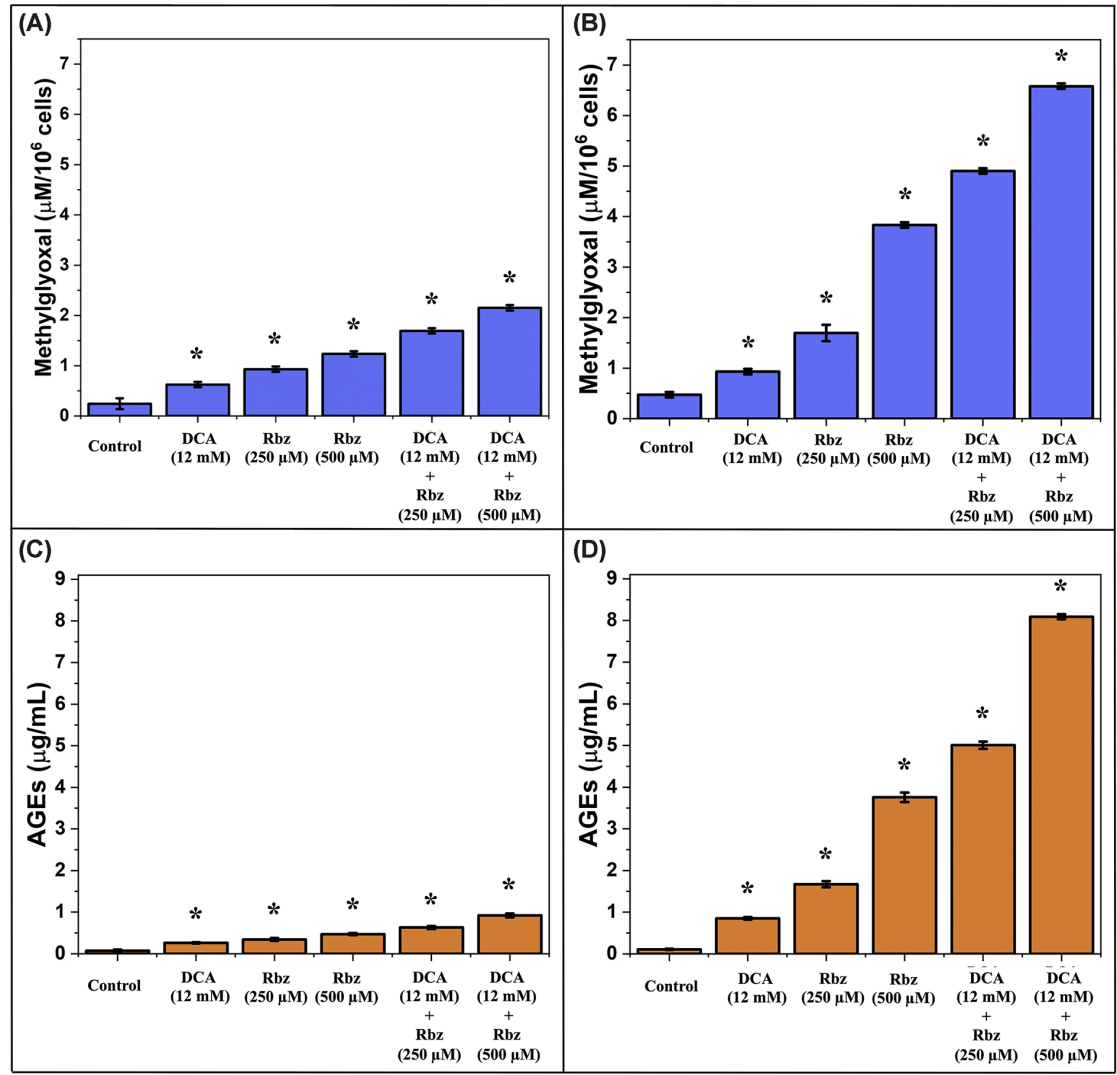
Quantification of MG and AGEs in normal and Jurkat T cells Normal T lymphocytes and Jurkat cells were treated with Rbz, DCA, or their combinations at the indicated concentrations. (**A**,**B**) MG levels and (**C**,**D**) AGEs levels were measured in normal (A,C) and Jurkat (B,D) cells after treatment. Data represent three independent biological replicates (mean ± SD). Statistical analysis was performed using one-way ANOVA followed by Tukey’s post-hoc test; statistical significance was defined as *P*<0.05 (*). Individual data points for all biological replicates are provided in Supplementary Figure S11 to illustrate distribution and variability.

The metabolic consequences of TPI inhibition were quantitatively assessed by measuring MG and advanced glycation end products (AGEs) following pharmacological treatment. In normal T lymphocytes, baseline MG levels were low (0.24 μM) and increased modestly with Rbz, reaching 0.93 and 1.24 μM at 250 and 500 μM, respectively (Figure 8A).

The highest MG accumulation in these cells was observed with combined DCA + Rbz at 500 μM, reaching 2.08 μM (∼8.7-fold increase). One-way ANOVA followed by Tukey’s post-hoc test confirmed that all treatment increments in normal lymphocytes were statistically significant compared with the control (*P*<0.05). In striking contrast, Jurkat cells exhibited substantially greater and significant MG accumulation under the same conditions (Figure 8B). Basal levels (0.32 μM) increased significantly to 1.69 and 3.68 μM with Rbz alone, and reached 6.50 μM with DCA + Rbz 500 μM (*P*<0.05), representing a ∼20-fold increase over control and more than triple the maximal value observed in normal lymphocytes.

The differential response was even more pronounced when examining AGEs formation. In normal lymphocytes, AGEs increased significantly from 0.073 μg/ml in controls to 0.47 μg/ml with Rbz 500 μM, reaching a maximum of 0.92 μg/ml with DCA + Rbz 500 μM (*P*<0.05, Figure 8C). Jurkat cells, however, reached far higher values, increasing significantly from 0.109 μg/ml in controls to 3.76 μg/ml with Rbz 500 μM and 8.09 μg/ml with DCA + Rbz 500 μM (*P*<0.05, Figure 8D). This ∼148-fold increase in leukemic cells was nearly ninefold higher than the maximal value recorded in normal T lymphocytes.

The magnitude of carbonyl stress in Jurkat cells closely correlated with TPI inhibition (Figure 6D), as these cells showed ∼50% greater loss of enzyme activity than normal lymphocytes at equivalent drug concentrations. This relationship supports a mechanism in which modified TPI isoforms in Jurkat cells are preferentially inhibited by Rbz, leading to glycolytic disruption, accumulation of triose phosphates, and subsequent MG overproduction.

Significantly, the relatively modest increase in MG and AGEs in normal T lymphocytes, despite preserved TPI activity, suggests that Rbz may exert additional effects unrelated to its primary target in these cells.

### Combined therapy of DCA and rabeprazole selectively induces apoptotic cell death in leukemic cells

Finally, given the marked increase in MG and AGEs, flow cytometry assays were performed to assess whether cell death was driven by apoptosis. Annexin V and propidium iodide staining, well-established markers for distinguishing early and late apoptosis from necrosis, revealed striking differences in apoptotic and necrotic responses between normal T lymphocytes and Jurkat cells after treatment with DCA, Rbz, or their combination. The specificity of these markers was confirmed using apoptosis and necrosis controls (Supplementary Figure S12).

**Figure 9:**
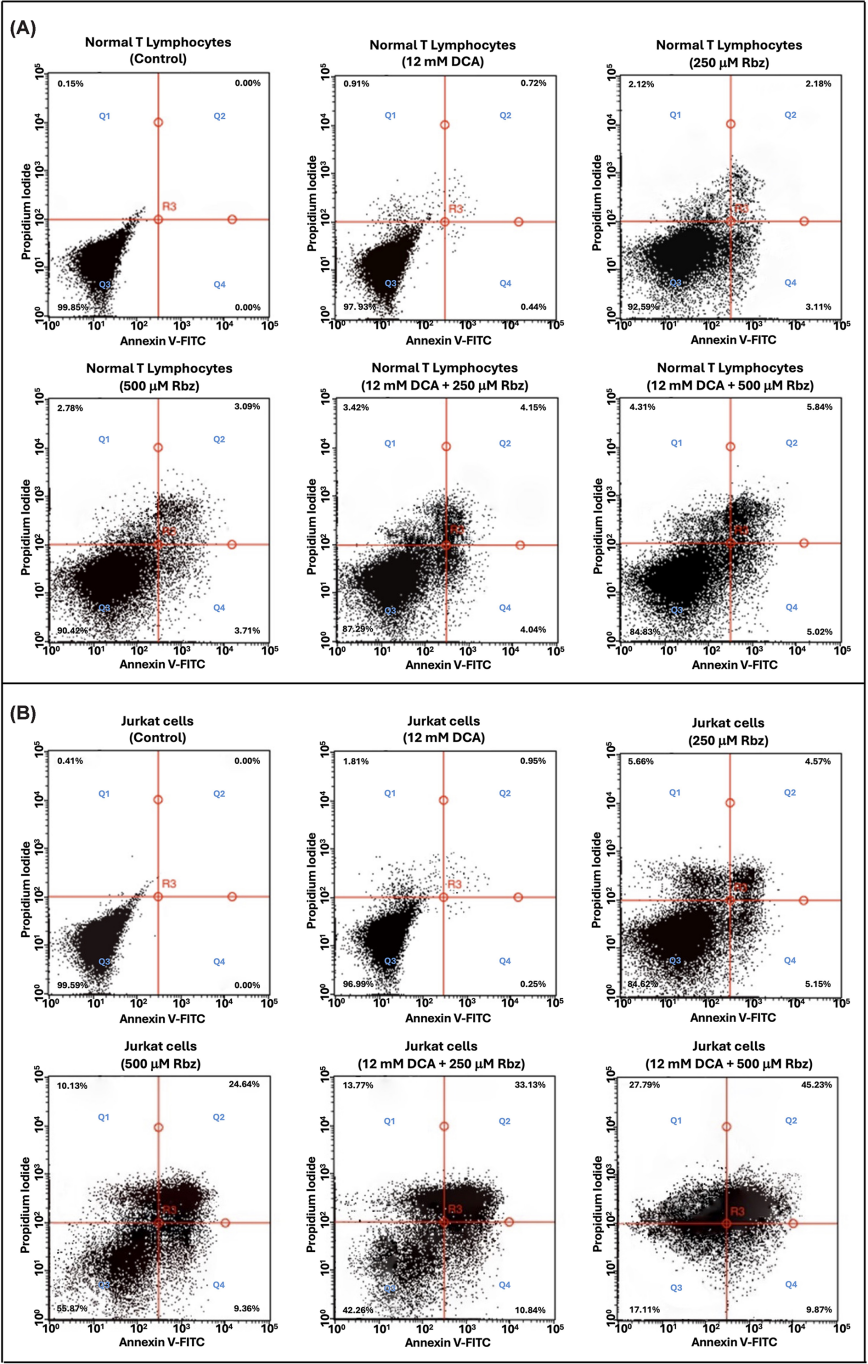
Flow cytometric analysis of apoptosis and necrosis in normal T lymphocytes (A) and Jurkat cells (B) treated with DCA, Rbz, or their combination Cells were exposed to 12 mM DCA, 250 μM or 500 μM Rbz, or pretreated with DCA for 24 h followed by Rbz for an additional 24 h. Annexin V/Propidium Iodide staining was used to distinguish viable (Q3), early apoptotic (Q4), late apoptotic (Q2), and necrotic (Q1) populations. (**A**) Normal T lymphocytes maintained high viability across treatments, with minimal apoptotic or necrotic induction. (**B**) Jurkat cells displayed a dose-dependent increase in apoptosis and necrosis, most pronounced with DCA + Rbz 500 μM, which reduced viability to 17.1% and markedly elevated late apoptosis and necrosis. Data are representative of 100,000 cells analyzed in two independent experiments.

In normal T lymphocytes, viability remained consistently high across all treatments, with minimal induction of apoptosis or necrosis (Figure 9A and Supplementary Table S4). Control cells displayed near-complete viability (99.8%) and negligible apoptosis. DCA (12 mM) alone had a scarce effect, maintaining viability above 97% with apoptotic events below 1.2%. Rbz at 250 and 500 μM caused a slight increase in apoptosis and necrosis, reducing viability to 92.5% and 90.4%, respectively. The DCA–Rbz combination produced a modest synergistic effect, with the highest doses (DCA 12 mM + Rbz 500 μM) lowering viability to 84.8% and increasing early (5.0%) and late (5.8%) apoptosis. Nonetheless, these effects were modest, indicating that normal T lymphocytes are largely resistant to DCA–Rbz-induced cytotoxicity.

In contrast, Jurkat cells exhibited a markedly different response, showing a progressive increase in apoptosis and necrosis with treatment (Figure 9B and Supplementary Table S4). Control cells maintained high viability (99.5%) with no detectable apoptosis. DCA alone caused only a slight reduction in viability (96.9%) and kept apoptotic events below 1.2%. Rbz at 250 and 500 μM induced a dose-dependent increase in early and late apoptosis, lowering viability to 84.6% and 55.9%, respectively. The most pronounced cytotoxicity occurred with combination treatments: DCA plus Rbz 250 μM reduced viability to 42.2%, with early (10.8%) and late (33.1%) apoptosis, while the highest dose (DCA 12 mM + Rbz 500 μM) further decreased viability to 17.1%, accompanied by early (9.8%) and late (45.2%) apoptosis and elevated necrosis (27.7%).

Taken together, the observed cell death patterns indicate that TPI inhibition by Rbz effectively triggers apoptosis in Jurkat cells. At the same time, DCA appears to sensitize these cells to apoptotic induction, likely by modulating key metabolic pathways and lowering the threshold for apoptotic commitment. The pronounced cytotoxic synergy observed with the combined DCA–Rbz treatment underscores the therapeutic potential of concurrently targeting altered metabolic and glycolytic nodes. This strategy, aimed at exploiting vulnerabilities associated with specific TPI isoforms, offers a promising avenue for the selective elimination of T-AAL cells and warrants preclinical investigation as a targeted metabolic adjuvant therapy.

## Discussion

A large body of evidence demonstrates that cancer cells undergo metabolic reprogramming to meet their heightened energy demands and sustain rapid proliferation [21]. This reprogramming involves key alterations in glucose metabolism (notably the Warburg effect), amino acid metabolism, and lipid metabolism [22], enabling cancer cells to support growth, evade apoptosis, and adapt to the tumor microenvironment.

To function effectively in the altered metabolic landscape, cancer cells dynamically regulate glucose metabolism by redirecting it toward various biosynthetic pathways through PTMs of relevant proteins, including phosphorylation, acetylation, palmitoylation, and others [23]. These PTMs potentially can alter protein 3D structure, enzymatic activity, and interactions, making them potential selective targets for anticancer therapies [24,25]. Therefore, studying PTMs is widely relevant to determine their potential as selective therapeutic targets in cancer cells.

In this study, we provide evidence that deamidation and phosphorylation in TPI could contribute to the formation of a promising drug target in T-ALL using Jurkat cells as a representative model.

### Recombinant and molecular docking analyses

Numerous chemical approaches have been developed to identify reactive and functionally relevant Cys residues [26]. Among them, Cys alkylation using DTNB has remained a widely employed method for over six decades, owing to its simplicity, reliability, and ease of implementation [27]. In this study, we found that both deamidation- and phosphorylation-mimicking variants of TPI (dTPI and pTPI) exhibit significantly greater reactivity toward DTNB than wtTPI. This reactivity reflects structural rearrangements that expose previously buried Cys thiols to the solvent, either through local unfolding or conformational shifts.

Consistent with this interpretation, molecular docking analyses revealed that dTPI and pTPI display preferential binding to PPIs relative to wtTPI. This enhanced binding is attributed to the formation of altered or expanded binding pockets, which increase ligand accessibility. These structural changes are likely a consequence of PTM mimics, which remodel the enzyme’s surface topology.

Given that Cys residues are among the most nucleophilic amino acids in proteins, they are frequent targets of covalent drugs used to treat cancer and other diseases. Therefore, the increased Cys accessibility observed in dTPI and pTPI not only highlights the structural plasticity induced by these modifications but also suggests the emergence of novel, druggable conformations with therapeutic potential.

Based on the increased accessibility of Cys residues in dTPI and pTPI, we successfully tested their reactivity and susceptibility to covalent inactivation. Among the PPIs evaluated, Rbz demonstrated the most potent inhibitory effect against the modified TPI isoforms. Notably, Rbz binding not only reduced enzymatic activity but also decreased thermal stability, suggesting a dual mechanism of action involving both active-site inhibition and structural destabilization.

This mechanistic insight is consistent with other research demonstrating that Rbz selectively inhibited the growth of human MLL-fusion leukemia cell lines (MV4-11 and MOLM-13), while wild-type MLL1 cells (K562) and non-leukemic cells (L02 and HUVEC) showed no damage. In that study, Rbz down-regulated the expression of downstream oncogenic targets Hoxa9 and Meis1 by inhibiting MLL1 HMT activity and disrupting the MLL1–WDR5 protein–protein interaction. Furthermore, other PPIs similarly inhibited MLL1–WDR5 interactions and selectively suppressed MLL-rearranged leukemia cell growth, with structure–activity relationship analyses underscoring the importance of PPI scaffolds for this inhibitory activity [28].

Moreover, recent studies identified Rbz and Ptz as potential FOXM1 inhibitors through pharmacophore-based virtual screening and molecular docking, with molecular dynamics simulations confirming stable interactions with FOXM1. *In vitro* assays demonstrated that Rbz inhibited FOXM1 at concentrations as low as 10 μM in BT-20 and MCF-7 breast cancer cells. In comparison, Ptz inhibited FOXM1 at 30 μM in BT-20 cells and at 70 μM in MCF-7 cells, leading to reduced cell proliferation [29].

These findings underscore the multimodal anticancer potential of PPIs such as Rbz, which can act through multiple, context-dependent mechanisms, including direct inhibition and destabilization of metabolic enzymes (as demonstrated for TPI), disruption of oncogenic epigenetic protein–protein interactions (MLL1–WDR5 in leukemia), and inhibition of transcription factors such as FOXM1 in breast cancer. The convergence of enhanced Cys accessibility, increased PPI binding affinity, enzymatic destabilization, and selective inhibition observed in modified TPI parallels mechanisms described for other oncogenic targets and supports the broader concept that PPIs can exploit PTM-driven conformational states to achieve selective protein targeting.

### Cellular studies and therapeutic implications

At the cellular level, Jurkat T-ALL cells were found to contain endogenous dTPI and pTPI isoforms, whose abundance increased upon pharmacological treatment, thereby exposing them as potential vulnerabilities within leukemic cells. Treatment with Rbz significantly reduced TPI activity and cell viability; importantly, co-treatment with DCA further potentiated this inhibitory effect, indicating that combined targeting of metabolic nodes enhances suppression of glycolysis and survival pathways.

Interestingly, similar mechanisms of PTM-dependent vulnerabilities have been reported in leukemia. For example, the histone methyltransferase G9a, which catalyzes H3K9me2/me3 modifications, undergoes automethylation at lysine residues distal to its active site. This methylation enhances G9a’s interaction with heterochromatin protein 1γ, forming a regulatory complex essential for gene expression modulation [30]. Importantly, increased G9a methylation has been shown to sensitize glucocorticoid-resistant B-ALL cells to treatment, highlighting how PTMs can create therapeutic entry points by modulating protein–protein interactions or enzyme activities critical for leukemic cell survival.

These observations complement the therapeutic repertoire by targeting post-translationally modified metabolic enzymes (e.g., TPI) and epigenetic regulators (e.g., G9a) as a strategy to overcome treatment resistance and exploit leukemia-specific vulnerabilities.

Our findings demonstrate a selective cytotoxic effect of Rbz in leukemic cells, closely correlated with the inhibition of TPI activity and the presence of modified TPI isoforms. Jurkat cells exhibited concentration-dependent sensitivity to Rbz, which was significantly enhanced by co-treatment with DCA, while normal T lymphocytes remained largely unaffected. The cytotoxic effects mirrored intracellular TPI inhibition under the same conditions, strongly supporting an on-target mechanism in which Rbz exerts its antileukemic action through TPI enzymatic modulation.

These findings reinforce the growing evidence that PPIs, including Rbz, possess antineoplastic properties that extend beyond their canonical role in gastric acid suppression. Reported mechanisms of action in cancer models encompass signaling pathway modulation, metabolic enzyme inhibition, and transcription factor targeting.

One line of evidence indicates that Rbz can inhibit ERK1/2 phosphorylation, producing antiproliferative effects in tumor cells [10]. Although ERK signaling was not examined in the present leukemia model, this pathway is known to intersect with metabolic regulation. Consequently, an indirect connection between ERK modulation and TPI inhibition cannot be excluded, and further studies are warranted to determine whether such cross-talk contributes to the metabolic vulnerability observed here.

Another reported mechanism involves the direct inhibition of the thioesterase domain of fatty acid synthase (FASN), resulting in the disruption of lipid biosynthesis and induction of apoptosis in cancer cells [31]. This is particularly relevant because FASN activity depends on metabolites generated upstream in glycolysis, where TPI contributes to the production of glycerol-3-phosphate and pyruvate, key precursors for lipogenesis. The present results suggest that Rbz may interfere with cancer metabolism at multiple metabolic nodes, with TPI inhibition representing a proximal checkpoint. This effect may be especially pronounced in leukemic cells enriched in accumulated or post-translationally modified TPI isoforms.

Rbz has also been described as an inhibitor of the transcription factor FOXM1, a central regulator of cancer cell proliferation and metabolic reprogramming, including the control of glycolytic flux [29]. Although FOXM1 was not directly assessed in this study, its regulation of numerous metabolic genes raises the possibility that TPI expression or modification could be downstream of FOXM1 activity. The observed accumulation of modified TPI isoforms in Jurkat cells, along with their heightened sensitivity to Rbz, supports the hypothesis that FOXM1-related pathways may influence the altered TPI landscape in leukemia.

More recently, chemoproteomic studies have revealed that Rbz can form covalent conjugates with Cys-rich, zinc-coordinating proteins, independently of acidic activation [32]. This mechanism offers a plausible explanation for direct TPI targeting, as TPI contains reactive cysteine residues that become more solvent-exposed or structurally destabilized following deamidation or phosphorylation. In the present system, treatment with DCA increased the abundance of such modified TPI isoforms in Jurkat cells, potentially facilitating Rbz activation and covalent binding. This zinc-independent, cysteine-targeted reactivity may also account for the selective sensitivity of leukemic cells, as these modified TPI isoforms are largely absent in normal lymphocytes.

Our results demonstrate that leukemic Jurkat cells harbor a distinctive spectrum of post-translationally modified TPI isoforms, not detected in normal T lymphocytes, that persists under basal conditions (without treatment) and increases following DCA and Rbz treatment. Analysis using nPAGE and phospho-specific immunoblotting revealed coexisting dTPI and pTPI forms, with combined DCA–Rbz exposure correlating with their greatest abundance and heterogeneity. In normal T lymphocytes, TPI remained unmodified regardless of treatment, confirming the Jurkat-specific presence of these PTMs. These findings extend our previous observations [6] and provide new insights into the dynamics of TPI PTMs in such leukemic model. While the close association between the induction of these isoforms and the subsequent loss of cell viability suggests a potential mechanistic link, these results provide a compelling biochemical framework for understanding how leukemia-specific TPI modifications may serve as targetable vulnerabilities in malignant metabolism.

The enrichment of acidic TPI variants, including those comigrating with mono- and doubly-deamidated controls, supports drug-induced deamidation and phosphorylation in Jurkat cells. Anti-phosphoserine blots showed abundance of pTPI following DCA–Rbz treatment. Therefore, such modifications are functionally relevant, as they correlate with heightened drug sensitivity and catalytic fragility, positioning these isoforms as selective therapeutic targets. This is consistent with recent reports [20] that identify dTPI as a cancer-specific vulnerability and a preferential target for thiol-reactive inhibitors. Our data extend this concept to model of leukemia, where both deamidation and phosphorylation appear to enhance drug binding, as suggested by *in vitro* assays using PTM-mimicking TPI recombinants.

The drug-induced phosphorylation observed here aligns with studies showing phosphorylation as a determinant of treatment responsiveness [33]. As in those models, phosphorylation of a central protein, such as TPI, emerges in a disease-specific manner and may act as an early marker of drug efficacy. Our results suggest that DCA may act upstream to induce TPI phosphorylation, with Rbz amplifying the effect through redox-mediated or conformational mechanisms that increase kinase accessibility.

The proposed capacity of PPIs to induce phosphorylation is further supported by evidence that Rbz enhances phospho-signaling, including GSK3β, ERK, and CREB pathways [34]. Although upstream kinases were not assessed in this study, the observed TPI phosphorylation may reflect a broader Rbz-induced shift in the phosphoproteome. The interplay between phosphorylation and deamidation could produce synergistic structural destabilization, yielding functionally distinct, drug-sensitive isoforms.

### Targeting TPI in Jurkat cells is associated with selective accumulation of MG and AGEs

Our findings reveal a strong correlation between TPI inhibition by Rbz and carbonyl stress selectively in the leukemic model, with a drastic accumulation of MG and AGEs observed. While normal T lymphocytes experienced significant but modest increases in MG and AGEs. This disparity is consistent with the preferential inhibition of modified TPI isoforms in leukemic cells, suggesting a model where glycolytic disruption leads to the accumulation and spontaneous degradation of triose phosphates into MG. Examples of this are previous studies showing MG toxicity in leukemic cells, particularly HL60 cells, where MG exposure inhibited proliferation and apoptosis [35].

The observed selective toxicity toward rapidly dividing malignant cells, in contrast to the relative sparing of mature peripheral leukocytes, correlates with the patterns of MG accumulation. This suggests that MG-induced stress may contribute to an inherent anti-leukemic effect, though the precise causative link remains to be fully established.

Importantly, our approach parallels the pathological conditions observed in TPI deficiency, where triose phosphate accumulation is associated with MG overproduction and systemic AGEs buildup [36]. In both contexts, impaired TPI function is linked to a toxic metabolite overflow, further supporting the association between this enzyme’s activity and the control of carbonyl stress. However, unlike the congenital TPI deficiency model, where detoxification mechanisms can potentially adapt gradually, the acute and targeted inhibition in our model likely overwhelms the glyoxalase pathway, especially in leukemic cells with pre-existing metabolic vulnerabilities.

Previous reports also underscore the anti-cancer potential of MG. For instance, glioblastoma cells exposed to exogenous MG exhibited apoptosis, proliferation arrest, and senescence [37]. However, achieving effective intracellular concentrations of MG through extracellular supplementation poses challenges due to detoxification and uptake limitations. In contrast, our results suggest that TPI inhibition is associated with a robust, cell-intrinsic generation of MG, bypassing the need for exogenous delivery and enabling what appears to be a more physiologically relevant and sustained stress signal. Such strategy may enhance therapeutic specificity while minimizing toxicity in non-cancerous cells.

Furthermore, MG accumulation has been implicated in Jurkat cell apoptosis via activation of the JNK pathway [38], a mechanism that may parallel the viability loss observed in our treated cells. Our findings are consistent with the hypothesis that MG acts not merely as a byproduct of metabolic disruption but potentially as a key factor in leukemic cell death. This aligns with a model in which carbonyl stress serves as a biomarker of target engagement and may contribute to the observed effector mechanisms, a dual role that warrants further investigation of its therapeutic relevance.

Finally, parallels can be drawn with recent work on butyrate in prostate cancer, where inhibition of the JAK2/Stat3/Nrf2/Glo1 pathway suppressed MG detoxification, resulting in cell death via MG accumulation [39]. Although our study does not directly assess Glo1 regulation, the striking accumulation of AGEs in Jurkat cells suggests a possible impairment or saturation of detoxification systems. The preferential TPI inhibition and MG accumulation in leukemic cells suggest that metabolic rewiring in cancer may sensitize cells to MG-mediated toxicity when glycolytic enzymes like TPI are perturbed.

Our results position TPI as a potential viable metabolic target in leukemia, where its inhibition appears to be associated with a cascade of metabolic stressors, including MG and AGEs. The observed selectivity for cancer cells, combined with the mechanistic data linking enzymatic inhibition to metabolic disruption, provides a strong rationale for further preclinical evaluation of TPI inhibitors such as Rbz as novel anti-leukemic agents.

### Rbz in combination with DCA is associated with apoptotic responses in leukemic cells and TPI inhibition

Our results show that combined treatment with DCA and Rbz is associated with robust apoptotic cell death selectively in leukemic Jurkat cells. In contrast, normal T lymphocytes displayed remarkable resistance to the same treatment.

This response pattern is consistent with previous reports that PPIs, such as Omz, induce apoptosis in leukemia-derived cell lines [40]. In that study, Omz promoted apoptosis in Jurkat cells through a mechanism involving both caspase activation and lysosomal destabilization, suggesting that PPIs can trigger cell death through multiple pathways. Similarly, our observed Rbz-induced apoptosis in Jurkat cells could involve lysosomal cathepsins in addition to caspase-dependent mechanisms, especially given the substantial late-apoptotic and necrotic populations observed at higher doses.

The observed selectivity may be associated with the acidic microenvironment characteristic of cancer cells, which is reported to favor PPI activation [41]. Once activated, PPIs have been shown to generate reactive oxygen species (ROS), leading to lysosomal and mitochondrial destabilization, membrane permeabilization, and cell death. Importantly, this ROS-driven apoptosis was reported to be caspase-independent, a phenomenon that could also be occurring in our system, particularly at higher Rbz concentrations, where necrotic features become prominent. The contribution of ROS may be synergistically enhanced by the glycolytic stress induced by TPI inhibition and MG accumulation, as shown in our earlier results, further sensitizing leukemic cells to oxidative and lysosomal stress.

In addition to ROS generation and lysosomal damage, recent evidence indicates that PPIs disrupt proteostasis by impairing protein degradation pathways, thereby sensitizing cancer cells to stress-induced apoptosis [42]. This mechanism may be particularly relevant in our system, where MG and AGEs accumulate in parallel with TPI inhibition, potentially overloading the protein quality control machinery. The unfolded protein response, which may be triggered by excessive protein carbonylation and glycation, might exacerbate cellular stress, ultimately leading to apoptotic collapse. Such a multifaceted mechanism involving TPI inhibition, ROS production, lysosomal permeabilization, and proteostasis disruption may collectively contribute to the potent and selective cytotoxicity observed in Jurkat cells.

When integrated with previous findings on PPI-induced apoptosis, our data support the idea that Rbz, though primarily used as a gastric acid suppressor, holds significant potential as an anti-leukemic agent, particularly when combined with metabolic disruptors like DCA that elevate MG and AGEs to toxic levels.

These insights warrant further exploration into the mechanisms of cell death and stress pathways potentially triggered by this combination therapy, including the roles of caspases, ROS, and lysosomal cathepsins, which may serve as both biomarkers of efficacy and therapeutic targets in leukemia. Additionally, a deeper investigation into the characteristics of these specific protein PTMs is necessary to determine their viability as starting points for identifying excellent therapeutic targets against cancer, including ALL.

## Future directions

A logical next step arising from our findings is the comprehensive chemoproteomic mapping of Rbz’s covalent targets in leukemia. While our data support TPI as a primary metabolic target, Rbz’s thiol-reactive nature and capacity to engage cysteine-rich proteins suggest that other functionally relevant proteins may be modified in parallel.

Another critical avenue is the preclinical validation of the DCA–Rbz combination in T-ALL xenograft models. Although our *in vitro* data strongly support selective anti-Jurkat activity, *in vivo* studies are essential to evaluate pharmacokinetics, drug activation in the tumor microenvironment, therapeutic efficacy, and safety profiles. Such models would also enable dynamic monitoring of MG, AGEs, and TPI PTM status in response to treatment, providing mechanistic biomarkers for a possible clinical translation.

Given that our results show an association between drug-induced TPI phosphorylation and cellular sensitivity, phosphoproteomic analyses are warranted to investigate the upstream kinase networks involved. Such studies would help clarify whether DCA and Rbz correlate with kinase signaling modulation or if they promote conformational changes in TPI that increase kinase accessibility. Mapping these networks may identify additional factors, either kinases or regulatory pathways, that potentially cooperate with TPI inhibition to enhance the observed cytotoxicity.

Together, these future directions outline a strategic roadmap for advancing PTM-based metabolic targeting of enzymes like TPI toward clinically viable leukemia therapies.

## Materials and methods

The reagents and materials used during the experimental procedures are detailed below. Unless otherwise specified, all additional chemicals and reagents referenced in the study were obtained from Sigma–Aldrich (St. Louis, MO, U.S.A.). Luria-Bertani (LB) broth and isopropyl β-D-1-thiogalactopyranoside (IPTG) were supplied by VWR Life Science Products (Pennsylvania, PA, U.S.A.). α-Glycerol-3-phosphate dehydrogenase (α-GDH) and reduced nicotinamide adenine dinucleotide (NADH) were purchased from Roche Diagnostics (Mannheim, Germany). Bio-Rad (Hercules, CA, U.S.A.) provided the immobilized metal affinity chromatography (IMAC) resin. Sephadex G-25 Fine was acquired from Amersham Biosciences (Amersham, U.K.), and Amicon Ultra centrifugal filters with a 30 kDa cutoff were sourced from Merck-Millipore (Billerica, MA, U.S.A.). Cell culture reagents, including fetal bovine serum (FBS), penicillin–streptomycin, and trypsin-EDTA, were procured from Invitrogen (Carlsbad, CA, U.S.A.).

### Mutagenesis, cloning, overexpression, and purification of TPI recombinant enzymes

wtTPI and dTPI were previously cloned, expressed, and purified as described elsewhere [6]. These recombinant constructs included a N-term His-tag and a Tobacco Etch Virus protease (TEVp) recognition site to facilitate subsequent purification and tag removal.

Since phosphorylation at serine 21 (S21) in TPI is a well-documented PTM known to significantly influence the enzyme’s structure and function [8,17], a phosphomimetic variant (pTPI) was generated by substituting serine with glutamic acid at position (S21E) using site-directed mutagenesis. The S21E mutation was introduced into the *wttpi* gene using site-directed mutagenesis via PCR amplification. Mutagenic primers used for this purpose were: forward 5′ CGGAAGCAGGAACTGGGGGA 3′ and reverse 5′ TCCCCCAGTTCCTGCTTCCG 3′ (the mutated codon is underlined). To enable efficient amplification and cloning, external primers corresponding to the universal T7 promoter and T7 terminator regions were also included in the PCR reactions. Amplification was carried out under standard cycling conditions as previously described for other human *TPI* variants [13].

Following amplification, both the PCR product containing the S21E mutation and the pET-3a-HisTEV expression plasmid were digested with *Nde*I and *Bam*HI restriction enzymes. The resulting fragments were ligated to generate the recombinant expression construct. The presence of the desired mutation and the integrity of the entire coding sequence were confirmed by DNA sequencing.

The resulting pTPI-pET-3a-HisTEV expression plasmid, and those encoding wtTPI and dTPI, were used to transform *Escherichia coli* BL21-CodonPlus-RIL competent cells for recombinant protein production. Overexpression and purification were performed according to established protocols [6]. Briefly, transformed cells harboring the respective *tpi* constructs in the pET-3a-HisTEV plasmid were cultured at 37°C until reaching an optical density (OD) of 0.8 measured at 600 nm (OD600), at which point protein overexpression was induced with 0.4 mM IPTG and continued overnight at 30°C. Cells were then harvested, lysed, and the His-tagged TPI variants were purified by IMAC using a resin pre-equilibrated with 50 mM Tris–HCl pH 8.0, 150 mM NaCl.

Following purification, the His-TEV tag was removed by overnight digestion with TEVp (1:50, TEVp:TPI) in the presence of 1 mM dithiothreitol (DTT) at room temperature. Tag-free TPIs were subsequently recovered by IMAC to remove undigested protein and the protease, then concentrated using Amicon Ultra centrifugal filters (30 kDa cutoff). Proteins were further purified by precipitation with 75% ammonium sulfate and stored at 4°C until use. Before experimental assays, recombinant enzymes were equilibrated in 100 mM triethanolamine buffer at pH 7.4 (TEA) and pre-incubated at 4°C for 1 h with 5 mM DTT. The reducing agent was removed by passing the sample through a 1 ml Sephadex G-25 fine resin column pre-equilibrated in TEA buffer. Protein concentrations were estimated by absorbance at 280 nm using an extinction coefficient of ε = 33,460 M^−1^ cm^−1^. Purity and structural integrity were verified by SDS–PAGE (16%) followed by colloidal Coomassie Brilliant Blue staining.

### Acid activation of PPIs

To perform enzyme inactivation assays, 200 mM stock solutions (dissolved in dimethyl sulfoxide) of the following PPIs were freshly prepared immediately before use: Lsz, Esz, Ptz, Omz, and Rbz.

For acid activation, each 200 mM PPI stock solution was prepared in a mixture of 90% solvent and 10% 0.1 N HCl and incubated at room temperature in the dark for 3 h. After incubation, acid-activated PPIs were diluted to a final concentration of 2 mM for use in subsequent assays. The final solvent concentration in all working solutions was kept below 0.5% to avoid solvent-related effects on protein activity.

### TPI enzymatic activity and kinetic parameters determination of pTPI

The enzyme activity of all TPIs and kinetic parameters of pTPI were assessed in the direction of GAP to DHAP conversion using a coupled enzyme assay, as previously described [43], with minor modifications. In this assay, α-GDH served as the coupling enzyme. For each mole of DHAP produced by TPI, one mole of NADH is oxidized by α-GDH. NADH consumption was monitored at 340 nm using a Cary 50 UV-Vis spectrophotometer. Reactions were carried out in a total volume of 1 ml containing TEA buffer, 0.2 mM NADH, 0.9 U of α-GDH, and 1 mM GAP.

For kinetics assays, GAP concentrations ranged from 0.025 to 7 mM. Reactions were initiated by the addition of 5 ng/ml of purified wtTPI and pTPI, whereas 50 ng/ml of dTPI was used, previously equilibrated at room temperature. All assays were performed at 25°C, and initial velocities (*V*_o_) were recorded within the first 5 min of the reaction.

The velocity data were plotted as a function of substrate concentration. Kinetic parameters (*K*_M_ and *V*_max_) were determined by fitting the data to the Michaelis–Menten equation described elsewhere.

### Inactivation assays of wtTPI, dTPI, and pTPI with PPIs

To evaluate the susceptibility of wtTPI, dTPI, and pTPI to inactivation by PPIs, each enzyme was incubated at a final concentration of 0.2 mg/ml in TEA buffer in the absence or presence of 500 μM of Omz, esz, Ptz, Lsz, or Rbz. Incubations were carried out at 37°C for 2 h. Following incubation, aliquots were withdrawn and immediately assayed for residual enzymatic activity using the standard GAP-to-DHAP conversion assay. Enzyme activity measured after incubation without any PPI was defined as 100%, and all results were expressed as percent residual activity relative to the control. All enzymes and PPI solutions were prepared fresh and diluted immediately before use.

### Quantification of derivatized cysteine residues in TPI variants treated with rabeprazole

To assess the extent of Cys derivatization in wtTPI, dTPI, and pTPI following treatment with Rbz, each enzyme variant was incubated at a final concentration of 0.2 mg/ml in TEA buffer in the absence (control) or presence of 500 μM Rbz for 2 h at 37°C.

After incubation, aliquots were withdrawn for residual activity measurements using the standard enzymatic assay. Immediately thereafter, excess Rbz was filtered using Microcon 30 kDa centrifugal filters (Merck-Millipore) to remove unbound compound. The protein concentration of the filtered samples was recalculated based on absorbance at 280 nm.

The number of free thiols (non-derivatized Cys) remaining in the protein was determined spectrophotometrically using 5,5′-dithiobis-(2-nitrobenzoic acid) (DTNB). Assays were carried out in TEA buffer supplemented with 1 mM DTNB and 5% SDS to ensure complete exposure of Cys residues. The formation of 2-nitro-5-thiobenzoate anion (TNB^2−^) was monitored by measuring the increase in absorbance at 412 nm, using a molar extinction coefficient of ε_412_ = 13.6 mM^−1^·cm^−1^ [27]. The number of derivatized Cys residues was calculated by subtracting the free Cys of the derivatized enzyme from the free Cys of the control enzyme.

### Melting temperature (*T*_m_) determination of TPI variants

Thermal unfolding studies were conducted to evaluate the thermal stability of wtTPI, dTPI, pTPI, either untreated or treated with 200 μM Rbz.

Protein samples were first incubated at 37°C for 2 h, either in the absence or presence of Rbz. Following incubation, samples were filtered using Microcon 30 kDa centrifugal filters to remove unbound compound. Protein concentrations were then determined and adjusted to 0.2 mg/ml in 25 mM phosphate buffer (pH 7.4).

Thermal denaturation assays were performed using a Jasco J-810 spectropolarimeter equipped with a Peltier temperature control system and a 0.1 cm pathlength quartz cuvette. Protein unfolding was monitored by recording the circular dichroism spectrum at 222 nm, which reflects α-helical content. The temperature was gradually increased from 30°C to 80°C at a rate of 1°C/min^−1^. From the experimental data, the apparent fraction of denatured protein was calculated, and the Tm was determined, as previously reported [44].

### 3D structural modeling and molecular docking of TPI variants with PPIs

To evaluate the interaction between TPI and PPIs, molecular docking analyses were performed using available and modeled protein structures. The crystallographic structures of wtTPI (PDB ID: 2JK2) [16] and dTPI (PDB ID: 4UNK) [13] served as structural references. A 3D structural model of pTPI was generated using AlphaFold asisted with ColabFold2 [18,19], with the 4UNK structure used as a modeling template to ensure structural consistency across variants. The chemical structures of the selected PPIs were retrieved from the PubChem database (https://pubchem.ncbi.nlm.nih.gov/). Molecular docking simulations were performed using the CB-Dock server [45], which automatically identifies surface-accessible cavities, the largest of which corresponds to the interface between subunits in TPI, and ranks ligand poses based on predicted binding affinity. For each TPI structure, the top five docking poses for each PPI were obtained using the server’s default parameters, enabling a comparative analysis of potential binding modes and affinities across the wtTPI, dTPI, and pTPI forms.

### Assays in normal T lymphocytes and Jurkat cells with DCA and Rbz

To evaluate the effect of DCA and Rbz on normal T lymphocytes and the Jurkat lymphoblast cell line (TIB-152), comparative assays were conducted using both untreated (control) and pharmacologically treated cells under the following culture conditions.

First, were isolated normal T lymphocytes from healthy donors as follow. Peripheral blood was collected from three healthy adult volunteers aged 18–22 years at the National Institute of Pediatrics (Mexico City) in the first half of 2023. All procedures were conducted in accordance with the Declaration of Helsinki and approved by the Institutional Research, Biosafety, and Ethics Committees (protocol number: 2022/067). Written informed consent was obtained from all participants.

Each donor provided 50 ml of blood, and mononuclear cells were isolated via density gradient centrifugation using Lymphoprep^™^ Density Gradient Medium (STEMCELL Technologies, Germany GmbH, Cologne, Germany). Samples were centrifuged at 2000 rpm for 20 min, and the interface layer containing mononuclear cells was collected and resuspended in 1 ml of PBS. T lymphocytes were isolated using magnetic separation with anti-CD3-conjugated magnetic nanoparticles (MACS, Miltenyi Biotec, San Diego, CA, U.S.A.) and processed through an AUTOMACS system to separate the CD3^+^ T-cell fraction.

Second, Jurkat E6-1 cells were obtained from the American Type Culture Collection (ATCC, Rockville, MD, U.S.A.) and cultured in RPMI-1640 medium (Sigma–Aldrich, St. Louis, MO, U.S.A.) supplemented with 10% (v/v) fetal bovine serum, 2 mM L-glutamine, 1 mM sodium pyruvate, 100 U/ml penicillin, and 100 μg/ml streptomycin. Freshly isolated normal T lymphocytes were cultured under the same conditions, as RPMI supports their expansion upon anti-CD3 stimulation.

For all experiments, Jurkat cells from passages 2 to 5 and freshly isolated normal T lymphocytes were used. Cultures were maintained at 37°C in a humidified atmosphere containing 5% CO_2_. Prior to treatment, cells were centrifuged at 2500 rpm for 5 min, washed three times with PBS, and resuspended at a density of 1 × 10^5^ cells per well in six-well plates with a final volume of 1 ml.

Once the cell types were obtained, the individual and combined effects of DCA and Rbz, were evaluated, so cells were treated under the following conditions. For single-agent treatments, cells were exposed to either 12 mM DCA and 250- or 500 μM of Rbz for 24 h. To assess potential synergic effects, combined treatments included 12 mM DCA with either Rbz at 250 μM or 500 μM, and these were incubated for 48 h. All treatments were carried out in the same culture medium and under identical incubation conditions. Control cells received no active compounds but were treated with equivalent volumes of solvent to control for vehicle-related effects.

After treatment, cells were centrifuged, washed three times with PBS, and resuspended. Cell density was determined using a Neubauer hemocytometer, and cell viability was assessed via the MTT assay. For this, 1 × 10^3^ cells were seeded per well in 96-well plates in 100 μl of PBS. MTT reagent (final concentration of 0.5 mg/ml) was added, and plates were incubated at 37°C in the dark for 4 h. Formazan crystals formed during this period were solubilized in DMSO, and absorbance was measured at 570 nm using an Epoch microplate spectrophotometer (BioTek, Winooski, VT, U.S.A.).

Viability results were expressed as a percentage relative to the untreated control, which was considered to have 100% viability. Each experimental condition was assayed in triplicate across three independent experiments.

To assess cellular TPI activity, cells from each condition were harvested post-treatment, washed with PBS, and subjected to five freeze/thaw cycles (10 s in liquid nitrogen, followed by 2 min at 37°C) to ensure complete lysis. Protein concentration in the lysates was quantified using the Bradford assay.

For enzymatic assays, 80 μg of protein extract was added to a total volume of 1 ml of the standard GAP-to-DHAP conversion reaction mixture, and NADH consumption was monitored at 340 nm using a UV-Vis spectrophotometer. TPI activity was reported as percentage of residual enzymatic activity relative to the untreated control, which was defined as 100% activity. All assays were performed in triplicate, and results represent the average of three independent biological replicates.

### Analysis of recombinant proteins and immunoprecipitated TPI isoforms from cell extracts by native gel electrophoresis and Western blot

To evaluate the presence and modification of TPI in cellular samples, native polyacrylamide gel electrophoresis (nPAGE) was performed, followed by immunodetection. Jurkat cells and normal T lymphocytes were cultured and subjected to the following conditions. Normal T lymphocytes were either maintained untreated (control) or exposed to 12 mM DCA for 24 h, followed by an additional 24 h of incubation with 500 μM Rbz. Jurkat cells were analyzed under four conditions: untreated (control), treated with 12 mM DCA alone for 24 h, or co-treated with 12 mM DCA followed by 24 h of incubation with either 250 μM or 500 μM Rbz. At the end of each treatment, cells were collected and lysed in cold RIPA buffer containing Protease/Phosphatase Inhibitor Cocktail (5872) (Cell Signaling Technology MA, U.S.A.) and centrifuged at 12,000 rpm for 20 min at 4°C. The supernatant was collected, and protein content was quantified using the Bradford assay. The protein extracts from each culture (100 μg of total protein extract per treatment) were immunoprecipitated with anti-human TIM (H-11) (Santa Cruz Biotechnology CA, U.S.A.) for 1 h at 4°C. After this, protein A/G agarose (Santa Cruz Biotechnology, sc-2003) was added to the mixtures, which were adequately resuspended and incubated overnight at 4°C. The mixtures were centrifuged at 2000 rpm for 3 min and washed seven times with PBS. The immunoprecipitation products were washed with 0.2 M glycine–HCl to separate the protein A/G plus protein–antibody–protein complex. To obtain the isolated cellular HsTIM, samples were centrifuged at 2000 rpm for 5 min, immediately after which the supernatant was taken, and the pH was neutralized with Tris buffer (pH 8.0). For native gel analysis, 10 μl of each immunoprecipitated protein was loaded per lane onto a 7% nPAGE gel prepared in Tris-glycine buffer, pH 8.5. In parallel, 1 μg per lane of purified recombinant wtTPI, dTPI, and ddTPI were used as isoform markers; electrophoresis was conducted at a constant current of 7 mA for 3 h at 4°C.

Proteins were transferred to a PVDF membrane at 0.8 mA/cm^2^ for 1 h using a transfer buffer composed of 25 mM Tris, 192 mM glycine, and 20% methanol. After transfer, the membrane was blocked with TBS-T containing 5% BSA for 1 h at room temperature and incubated overnight at 4°C with a mouse anti-TPI monoclonal antibody (H11) diluted 1:1000 in TBS-T with 1% BSA. Following three washes with TBS-T, membranes were incubated with HRP-conjugated anti-mouse IgG secondary antibody (1:3000 dilution), and detection was performed using Clarity^™^ Western ECL substrate (Bio-Rad). Signal acquisition was performed using a ChemiDoc XRS+ system (Bio-Rad Laboratories, Hercules, CA, U.S.A.).

After detection with anti-TPI, the same membrane was stripped and re-probed using an anti-phosphoserine antibody (4A3) to assess the presence of serine-phosphorylated TPI isoforms. The same immunodetection procedure described above was followed.

To assess drug-induced conformational or charge alterations in TPI recombinant variants, 0.2 mg/ml of wtTPI, dTPI, and pTPI were incubated for 2 h at 37°C under the following conditions: no treatment (control), 12 mM DCA, or 12 mM DCA + 500 μM Rbz. After incubation, 10 μg of each sample was loaded on a 7% native gel and run under the same electrophoretic conditions described above.

After electrophoresis, gels were stained with colloidal Coomassie Brilliant Blue to visualize band migration and assess potential mobility shifts due to PTMs or drug-induced conformational changes. All experiments were performed in duplicate.

### Quantification of intracellular MG and AGEs

To evaluate the effects of DCA and Rbz on MG levels and the accumulation of AGEs, normal T lymphocytes and Jurkat cells were cultured and treated under the following conditions. Cells were either left untreated (control) or exposed for 24 h to 12 mM DCA, 250 or 500 μM Rbz, or a combination of 12 mM DCA with either 250 or 500 μM Rbz for 48 h. All treatments were performed under standard culture conditions. Following incubation, 5 × 10^6^ cells were harvested for further analysis.

Intracellular MG levels were determined spectrophotometrically using 2,4-dinitrophenylhydrazine (DNPH) as previously described, with slight modifications [6]. Cells were washed in PBS and lysed by five cycles of freezing and thawing. Afterward, 0.45 M perchloric acid was added, samples were incubated on ice for 10 min, and centrifuged at 12,000 rpm for 10 min at 4°C. Supernatants were collected and stored at −70°C until analysis. For standard curve generation, known concentrations of MGO (0–10 μM) were reacted with 0.2 mM DNPH in HCl–ethanol (12:88) at 42°C for 45 min. After cooling, absorbance was measured at 432 nm using a microplate spectrophotometer (Epoch, BioTek, U.S.A.). Sample MGO concentrations were calculated using the extinction coefficient (ε = 33,600 M^−1^ cm^−1^) and reported as μM MGO per 1 × 10^6^ cells. All assays were performed in triplicate.

AGEs were quantified using a commercial ELISA kit (MyBioSource, San Diego, CA, U.S.A.) following the manufacturer’s protocol. After treatment, cells were lysed in RIPA buffer with protease inhibitors. Total protein concentration was measured by the Bradford assay and adjusted to 1 mg/ml. Each sample was diluted 1:100, and AGE levels were determined via ELISA. Avidin–peroxidase conjugates and TMB substrate were used for color development, and absorbance at 450 nm was measured within 10 min using a microplate reader (Epoch, BioTek, U.S.A.). A standard curve (0–200 ng/ml) from the kit was used for quantification. Results are expressed as μg AGEs/ml and represent the mean of three independent experiments.

### Flow cytometry-based apoptosis assays

To assess apoptosis induction in response to pharmacological treatment, normal T lymphocytes and Jurkat cells were cultured in six-well plates at a density of 1 × 10^6^ cells per well in 2 ml of RPMI-1640 medium supplemented with 10% fetal bovine serum and antibiotics. Experimental treatments were designed to evaluate both the individual and combined effects of DCA and Rbz on cell viability and cell death pathways. In all assays, treatments were carried out for a total of 48 h, either as a single continuous exposure or in a sequential manner, depending on the experimental condition.

Cells were initially exposed to 12 mM DCA for 24 h. After, DCA-containing medium was replaced with fresh medium containing either 250 or 500 μM Rbz, and cells were incubated for an additional 24 h. In parallel, cells were also subjected to single-agent exposures with 250 or 500 μM Rbz for 24 h, or to continuous 48 h exposure with 12 mM DCA alone. Vehicle controls were included to account for potential effects of the solvent used for Rbz preparation, which was maintained at a final concentration below 0.5%.

At the end of the incubation period, cell density was assessed using a Neubauer counting chamber, and cells were harvested for apoptosis analysis. To distinguish between viable, early apoptotic, late apoptotic, and necrotic populations, cells were stained with Annexin V-FITC and propidium iodide (PI), according to the manufacturer’s protocol. Briefly, cells were resuspended at 1 × 10^6^ cells/ml in binding buffer, stained with Annexin V and PI, and incubated for 15 min at room temperature in the dark.

For accurate compensation and gating strategies, several control conditions were included: unstained cells, single-stained Annexin V or PI controls, and positive controls for apoptosis and necrosis. Apoptotic controls were generated by treating cells with 50 μM hydrogen peroxide (H_2_O_2_) for 5 h, while necrotic controls were obtained by exposure to 500 μM H_2_O_2_ for 5 h. These controls were used to build the compensation matrix and define quadrant boundaries in the dot plots.

Data acquisition was performed on a Guava^®^ easyCyte^™^ Flow Cytometer (Cytek^®^ Biosciences, Fremont, CA, U.S.A.), and at least 10,000 events per sample were recorded. Flow cytometry data were analyzed using InCyte^™^ Software v3.1 (Merck Millipore, Bedford, MA, U.S.A.). The percentages of cells in each quadrant (viable, early apoptotic, late apoptotic, and necrotic) were quantified and statistically analyzed across treatment groups to determine the extent of apoptosis induction and the potential enhancement of Rbz cytotoxicity by DCA co-treatment.

## Supplementary Material

Supplementary Figures S1-S12 and Tables S1-S4

## Data Availability

Data are contained within the article.

## Open Access

Open access for this article was enabled by the participation of Universidad Nacional Autonoma de Mexico (UNAM) in an all-inclusive *Read & Publish* agreement with Portland Press and the Biochemical Society.

## Abbreviations

α-GDH: glycerol-3-phosphate dehydrogenase
AGEs: advanced glycation end products
ALL: acute lymphoblastic leukemia
B-ALL: B-cell ALL
DCA: dichloroacetate
DHAP: dihydroxyacetone phosphate
DNPH: 2,4-dinitrophenylhydrazine
DTNB: 5,5′-dithiobis-(2-nitrobenzoic acid)
DTT: dithiothreitol
Esz: esomeprazole
FASN: fatty acid synthase
G3P: glyceraldehyde-3-phosphate
IMAC: immobilized metal affinity chromatography
Lsz: lansoprazole
MG: methylglyoxal
nPAGE: native PAGE
Omz: omeprazole
PDK: pyruvate dehydrogenase kinase
PPI: proton pump inhibitor
PTM: post-translational modification
Ptz: pantoprazole
Rbz: rabeprazole
ROS: reactive oxygen species
T-ALL: T-cell ALL
TPI: triosephosphate isomerase
