# Supplementary material for: Post-translational modifications of triosephosphate isomerase reveal metabolic vulnerabilities in T-ALL: effect of combining dichloroacetic acid and the PPI rabeprazole
Source: Biochem J. 2026 Feb 18;483(3):319–44. doi: 10.1042/BCJ20253451 (PMC13094655; doi:10.1042/BCJ20253451)
Supplement: Supplementary Figures S1-S12 and Tables S1-S4 [file BCJ-2025-3451_supp.pdf]

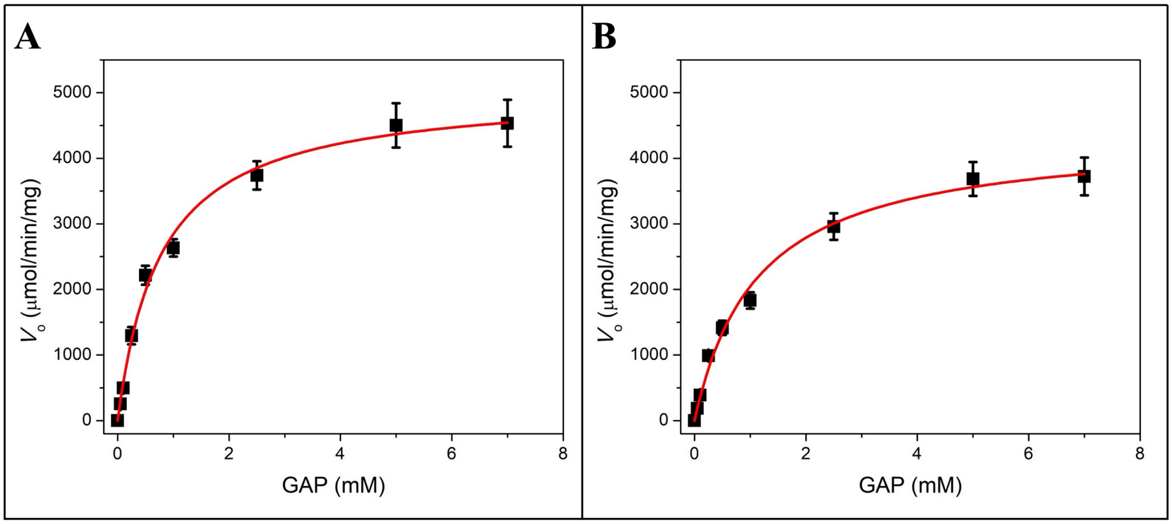

Supplementary Figure S1. Michaelis–Menten kinetics of recombinant wt and phosphorylation-mimicking TPI. Michaelis–Menten plots showing enzymatic activity of recombinant wtTPI (**A**) and the phosphorylation-mimicking mutant pTPI (S21E) (**B**) using GAP as substrate over a concentration range of 0–7 mM. Enzyme activity was measured using 5 ng/mL of purified protein under standard assay conditions. Data represent mean values obtained from three independent biological replicates.

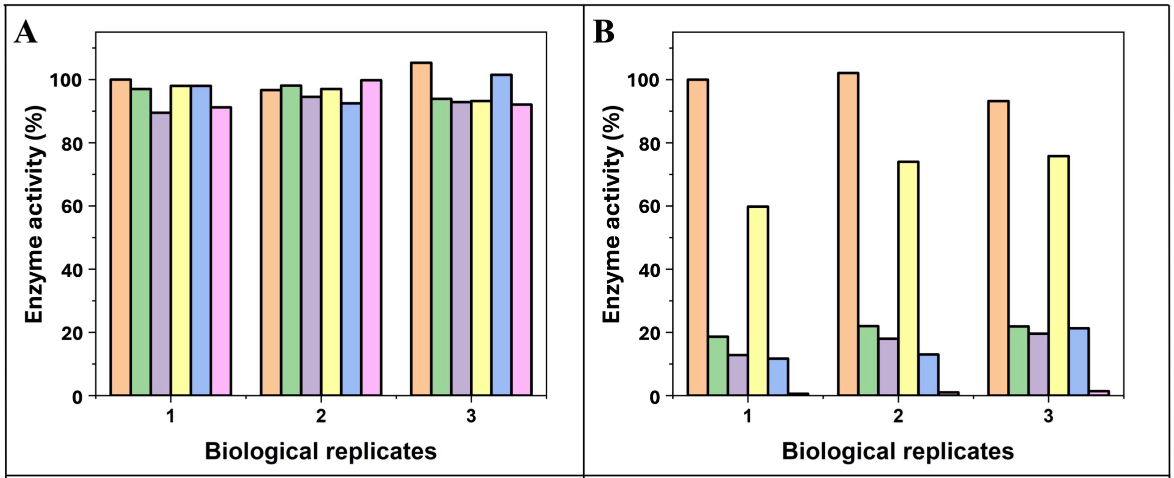

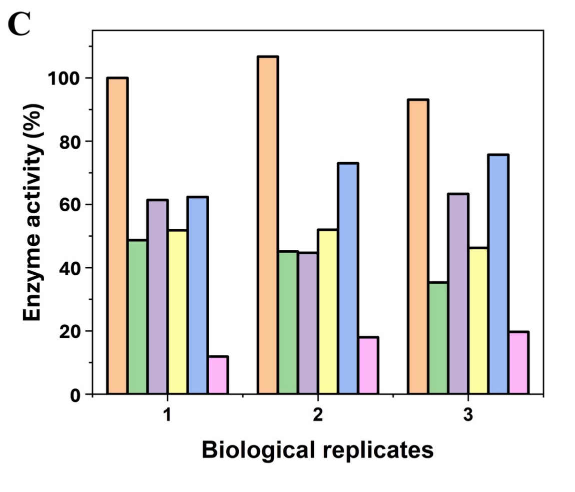

D

Enzyme	Biological replicate 1	Biological replicate 2	Biological replicate 3	Mean ± SD
**wtTPI**	0.90	0.82	0.68	0.80 ± 0.11
**dTPI**	2.75	2.99	2.95	2.90 ± 0.13
**pTPI**	2.32	2.05	2.53	2.30 ± 0.24

E

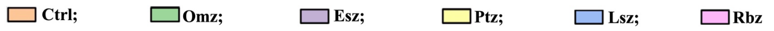

Supplementary Figure S2. Inactivation of WT and post-translational modification–mimicking TPI variants by PPIs. Enzymatic activity of recombinant wtTPI (**A**), dTPI (**B**), and pTPI (**C**) was assessed following incubation with PPIs at a final concentration of 500 µM. Panels **A**–**C** show individual biological triplicates corresponding to the experiments summarized in Figure 3 of the main manuscript. Panel **D** summarizes the extent of Cys derivatization by Rbz for each TPI variant, providing additional evidence of differential susceptibility to covalent modification. Panel **E** indicates the symbols used to represent the different PPIs in panels **A**–**C**.

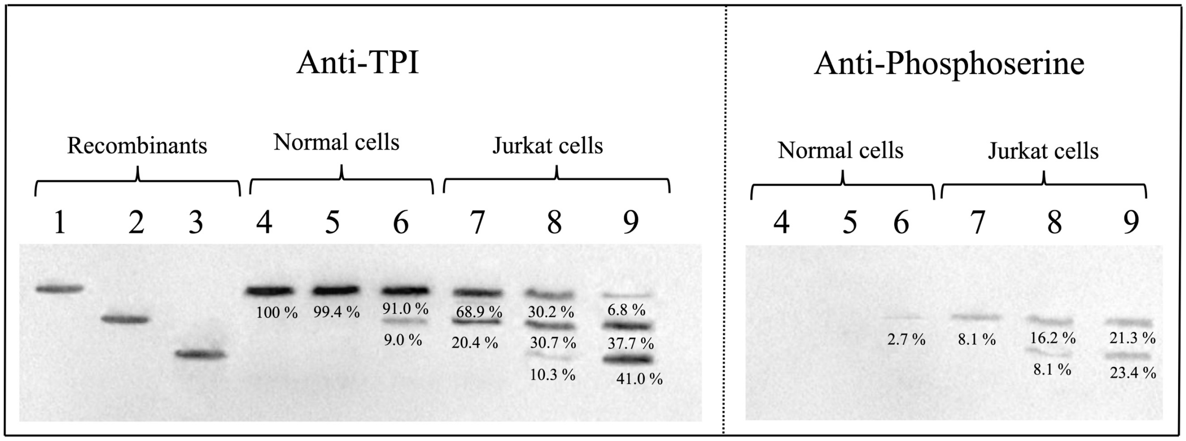

Supplementary Figure S3. Densitometric analysis of TPI isoforms and phosphorylation status in normal T lymphocytes and Jurkat cells under drug treatment. Left panel shows densitometric quantification of anti-TPI immunoblots corresponding to the nPAGE analysis presented in Figure 5A of the main manuscript. Right panel shows densitometric analysis of anti-phosphoserine immunoblots obtained from the same membrane following TPI immunoprecipitation. Purified recombinant wtTPI, dTPI, and ddTPI were used as migration references. Band intensities are expressed as percentages (indicated below each band) of total TPI and normalized to the intensity of the untreated normal T-cell sample (lane 4, left panel), which was set to 100%. Data represent mean densitometric values from two independent experiments.

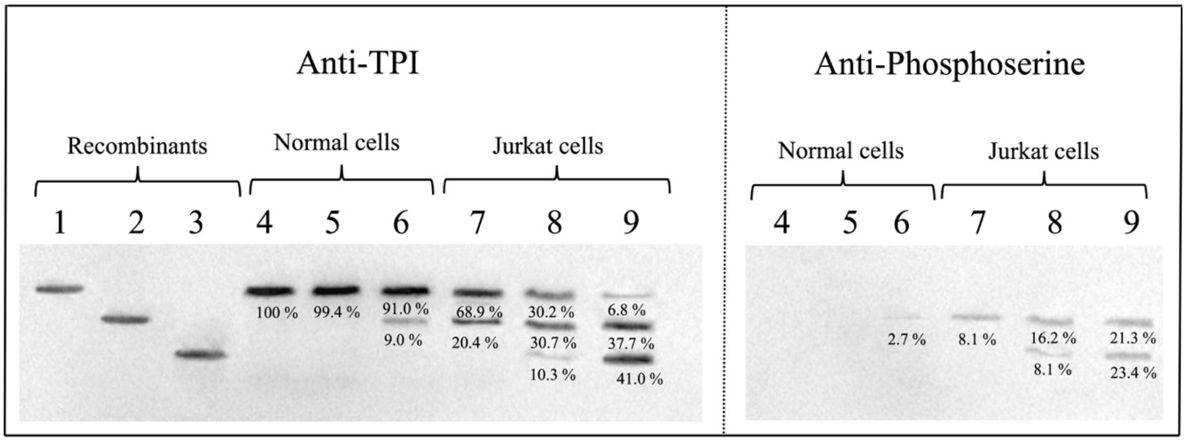

Supplementary Figure S4. Full-length nPAGE immunoblot of recombinant and cellular TPI isoforms. Full-length nPAGE blot corresponding to Figure 5 of the main manuscript. Anti-TPI immunoblot showing recombinant and cellular TPI isoforms. Lanes 1–3 contain 1 μg of recombinant wtTPI, dTPI, and ddTPI, used as migration standards. Lanes 4–6 contain TPI immunoprecipitated from normal T lymphocytes, and lanes 7–9 contain TPI immunoprecipitated from Jurkat cells. The positive and negative poles of the gel are indicated on the right. Under native conditions, proteins migrate according to their charge-to-mass ratio; the TPI monomer has an approximate molecular mass of 26.7 kDa and, as a native dimer, migrates with an apparent molecular mass of approximately 53.4 kDa.

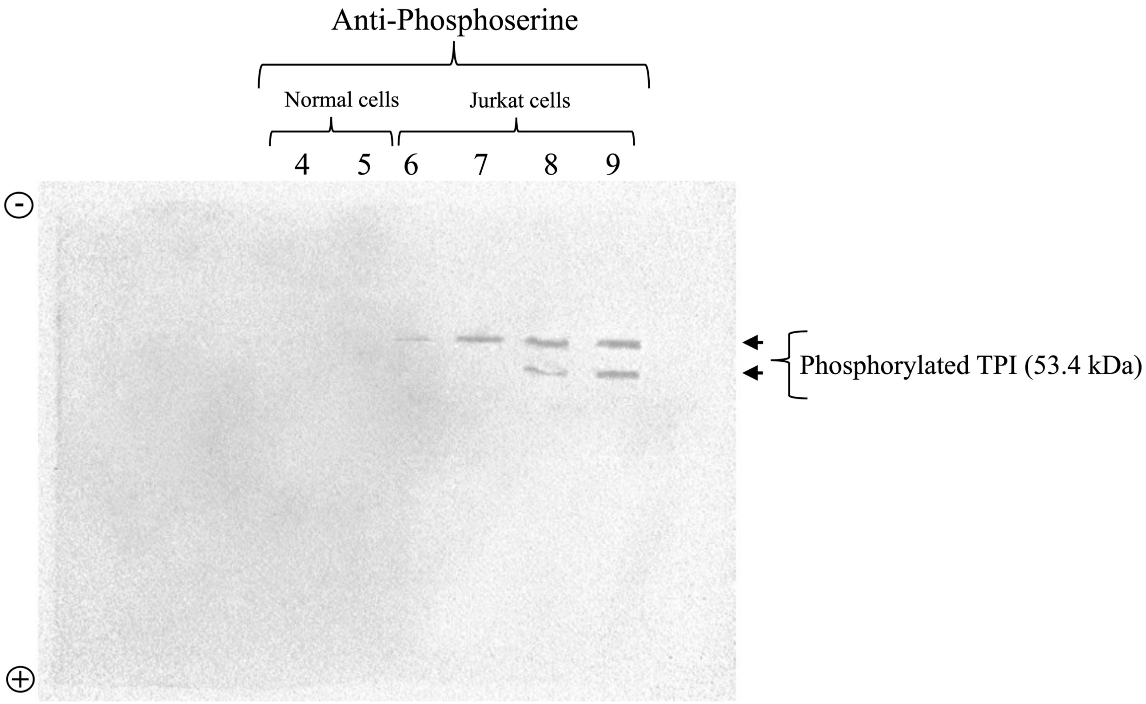

Supplementary Figure S5. Full-length nPAGE anti-phospho immunoblot of recombinant and cellular TPI isoforms. Full-length nPAGE blot corresponding to Figure 5 of the main manuscript. Anti-phospho immunoblot showing recombinant and cellular TPI isoforms. Lanes 1–3 contain 1 µg of recombinant wt TPI, dTPI, and ddTPI, used as migration standards. Lanes 4–6 contain TPI immunoprecipitated from normal T lymphocytes, and lanes 7–9 contain TPI immunoprecipitated from Jurkat cells. The positive and negative poles of the gel are indicated on the right. Under native conditions, proteins migrate according to their charge-to-mass ratio; the TPI monomer has an approximate molecular mass of 26.7 kDa and, as a native dimer, migrates with an apparent molecular mass of approximately 53.4 kDa.

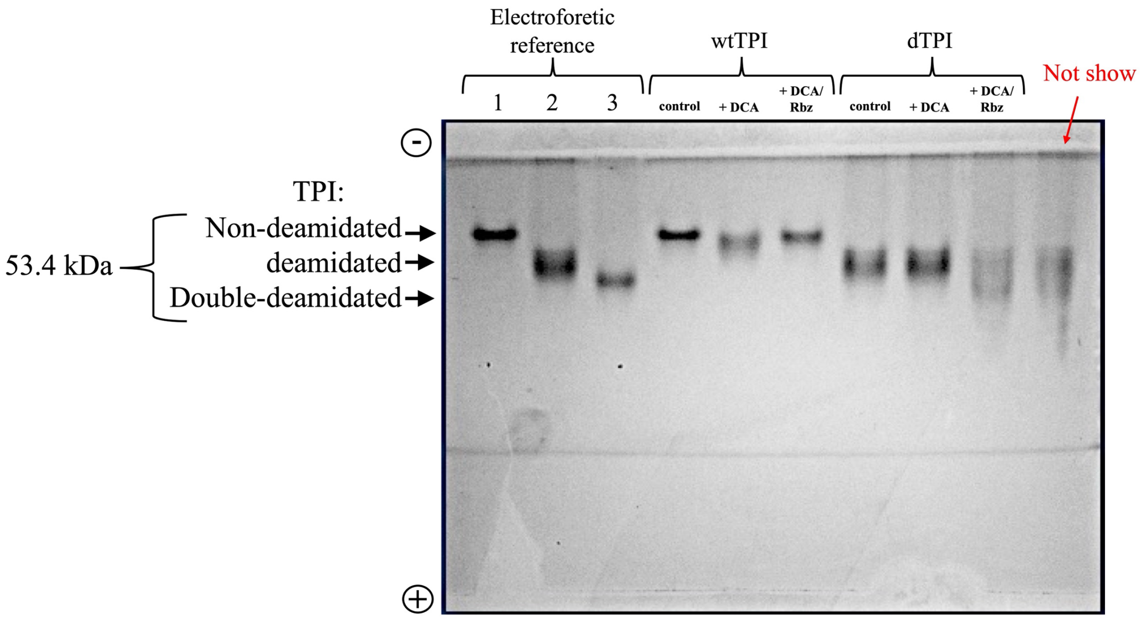

Supplementary Figure S6. Full-length Coomassie-stained nPAGE of recombinant WT and dTPI under drug treatment. Full-length nPAGE stained with Coomassie Brilliant Blue, corresponding to Figure 5**B** of the main manuscript. Lanes 1–3 contain recombinant wtTPI, dTPI, and ddTPI, used as migration standards. Lanes 4–6 contain wtTPI without treatment (control), incubated with DCA or the combination of DCA and Rbz, respectively. Lanes 7–9 contain dTPI subjected to the same treatments.

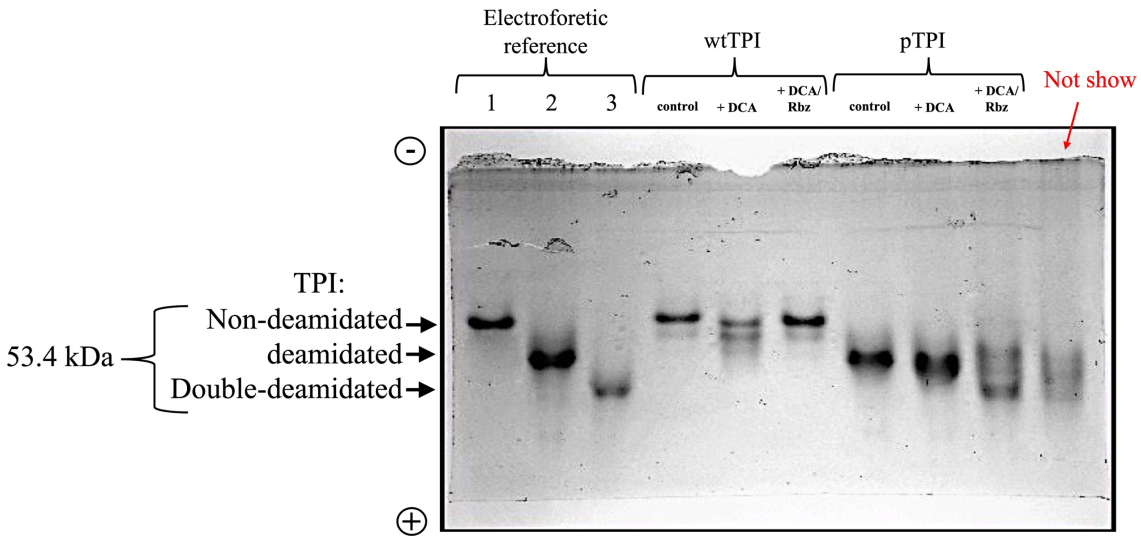

Supplementary Figure S7. Full-length Coomassie-stained nPAGE of recombinant WT and pTPI under drug treatment. Full-length nPAGE stained with Coomassie Brilliant Blue, corresponding to Figure 5**B** of the main manuscript. Lanes 1–3 contain recombinant wtTPI, dTPI, and ddTPI), used as migration standards. Lanes 4–6 contain wtTPI without treatment (control), incubated with DCA or the combination of DCA and Rbz. Lanes 7–9 contain pTPI subjected to the same treatments.

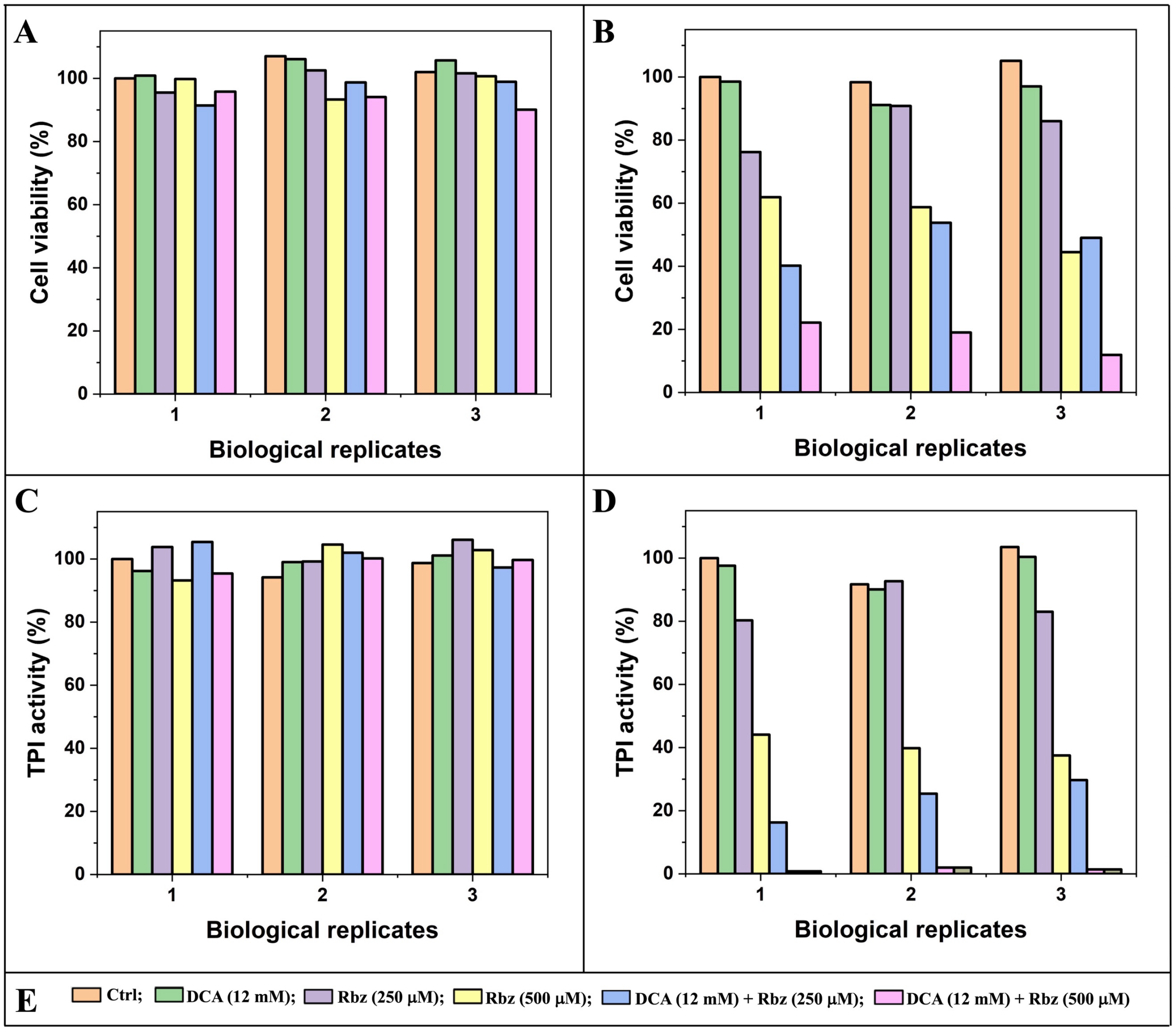

Supplementary Figure S8. Effects of DCA and Rbz on cell viability and endogenous TPI activity. Normal T lymphocytes and Jurkat T cells (1×10^5 cells per well) were incubated for 24 h under control conditions or in the presence of 12 mM DCA, Rbz (250 or 500 µM), or their combinations. Cell viability was assessed by MTT assay in normal T lymphocytes (**A**) and Jurkat T cells (**B**). Endogenous TPI catalytic activity was measured spectrophotometrically in normal T lymphocytes (**C**) and Jurkat T cells (**D**). Panels **A**–**D** show individual biological triplicates corresponding to the experiments summarized in Figure 6 of the main manuscript. Results are expressed as percentages relative to untreated controls, which were set to 100%. Panel **E** indicates the symbols used to represent the different treatments in panels **A**–**D**.

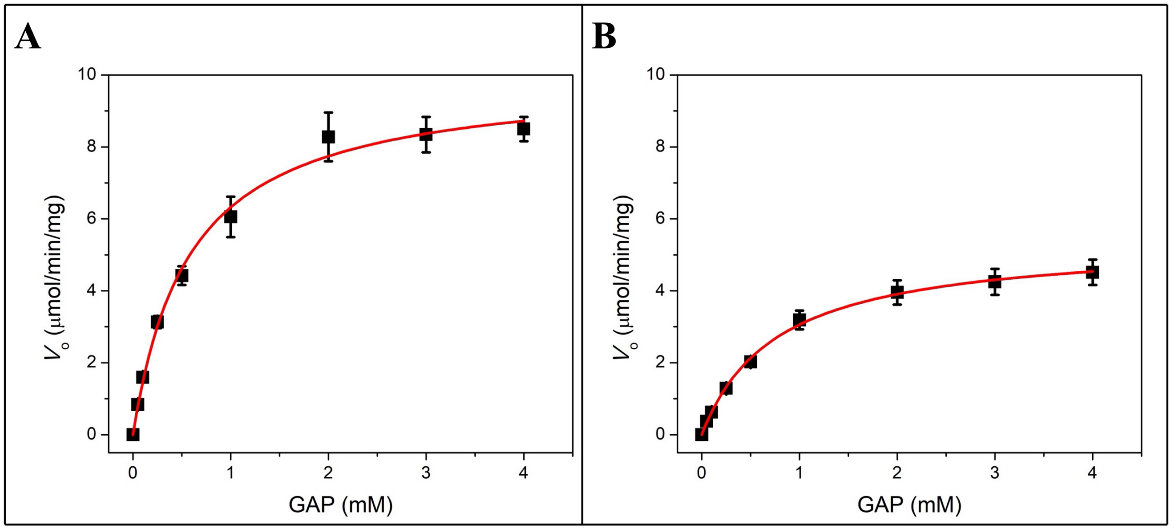

Supplementary Figure S9. Michaelis–Menten kinetics of endogenous TPI in Jurkat cell lysates under control conditions and Rbz treatment. Michaelis–Menten plots showing endogenous TPI activity in Jurkat cell lysates under control conditions (**A**) and following treatment with 6 mM Rbz (**B**). GAP was used as substrate over a concentration range of 0–4 mM, and enzyme activity was measured under standard assay conditions. Data represent mean values obtained from triplicate determinations.

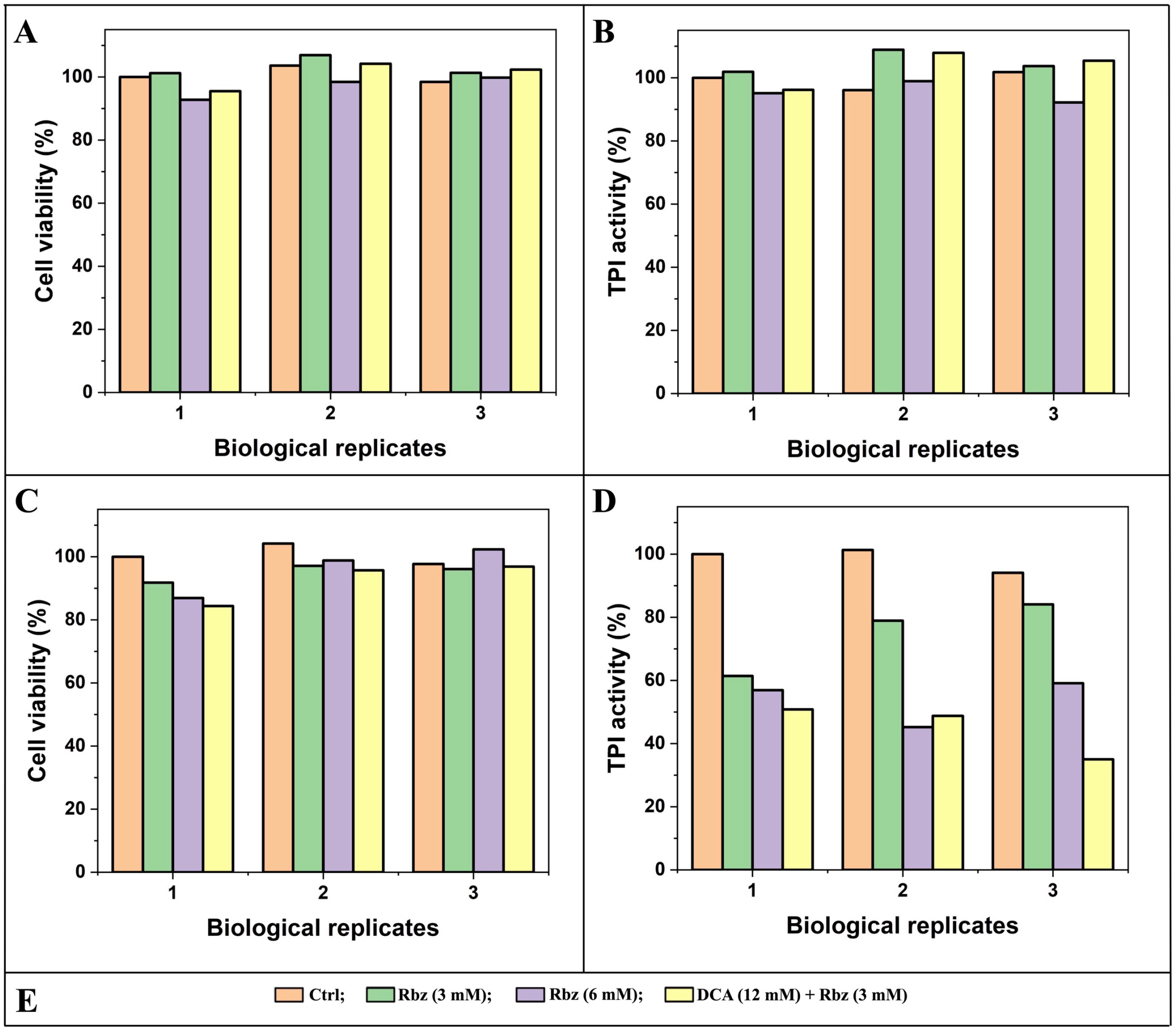

Supplementary Figure S10. Effects of acute Rbz exposure on cell viability and endogenous TPI activity. Normal T lymphocytes and Jurkat cells (1 × 10^5 cells per well) were exposed for 3 h under standard culture conditions to Rbz (3 or 6 mM) alone or to 12 mM DCA followed by 3 mM Rbz. Cell viability was assessed by MTT assay in normal T lymphocytes (**A**) and Jurkat cells (**C**). Endogenous intracellular TPI enzymatic activity was determined spectrophotometrically in clarified cell lysates from normal T lymphocytes (**B**) and Jurkat cells (**D**). Panels **A**–**D** show individual biological triplicates corresponding to the experiments summarized in Figure 7 of the main manuscript. Results are expressed as percentages relative to untreated controls, which were set to 100%. Panel **E** indicates the symbols used to represent the different treatments in panels **A**–**D**.

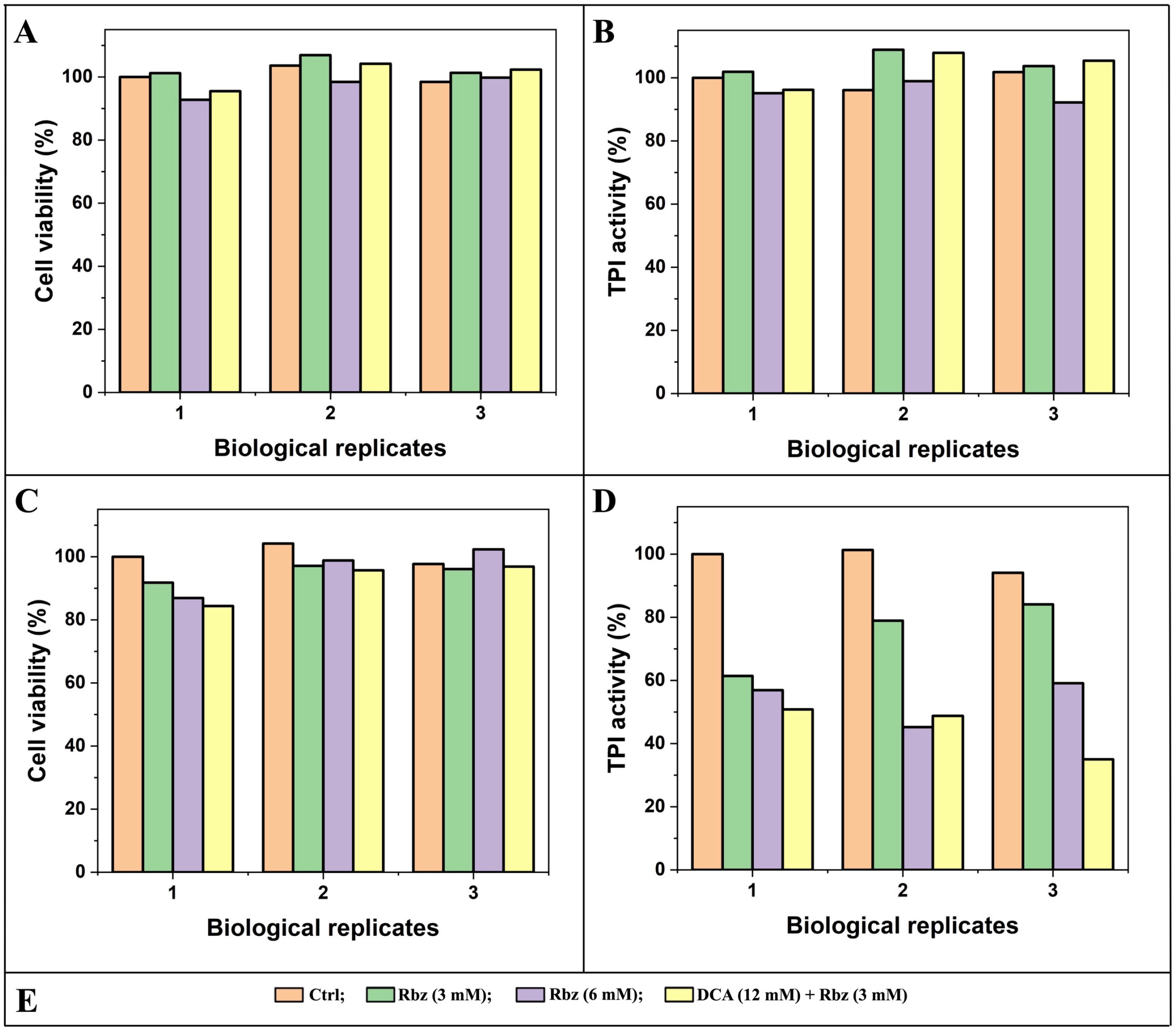

Supplementary Figure S11. Quantification of MG and AGEs in normal and Jurkat T cells following drug treatment. Normal T lymphocytes and Jurkat cells (1 × 10^5 cells per well) were treated under standard culture conditions with Rbz, DCA, or their combinations at the indicated concentrations. Intracellular MG levels were determined in normal T lymphocytes (**A**) and Jurkat cells (**B**). Levels of AGEs were quantified in normal T lymphocytes (**C**) and Jurkat cells (**D**) following treatment. Panels **A**–**D** show individual biological triplicates corresponding to the experiments summarized in Figure 8 of the main manuscript. Results are expressed relative to untreated controls, which were set to 100%. Panel **E** indicates the symbols used to represent the different treatments in panels A–D.

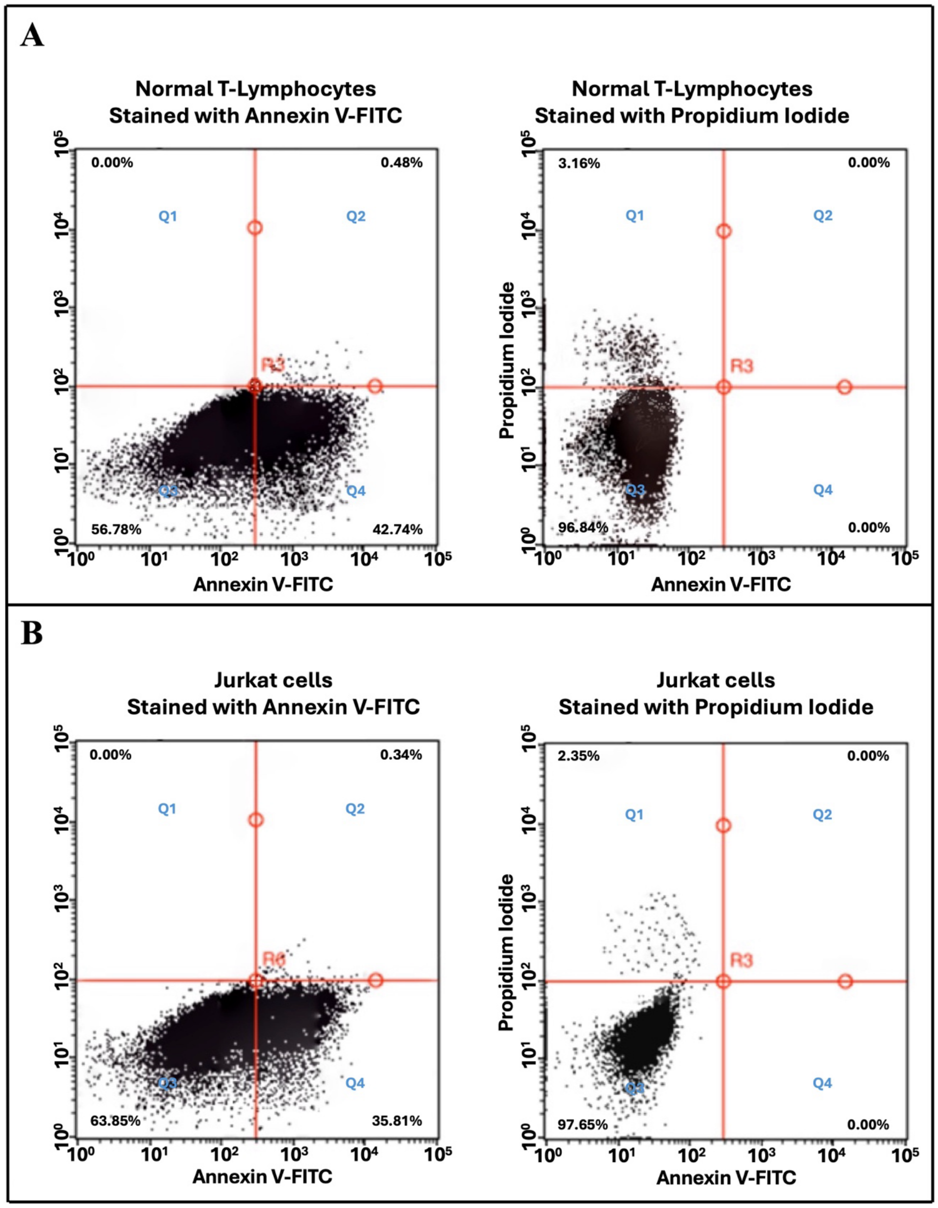

Supplementary Figure S12. Flow cytometric discrimination of apoptotic and necrotic populations in normal and Jurkat T cells. Representative flow cytometry plots illustrating the discrimination between apoptotic and necrotic cell populations in normal T lymphocytes and Jurkat cells. Cells were exposed to hydrogen peroxide (H_2O_2) at 50 µM to induce apoptosis (left panels in **A** and **B**) or 500 µM to induce necrosis (right panels in **A** and **B**) for 5 h at 37 °C. Cells were then washed, resuspended at 1×10^6 cells/mL, and stained with Annexin V and propidium iodide. Panel **A** corresponds to normal T lymphocytes and panel **B** to Jurkat cells. Quadrants indicate necrotic cells (Q1), late apoptotic cells (Q2), viable cells (Q3), and early apoptotic cells (Q4). Data are representative of 100,000 events collected from two independent experiments.

Supplementary Table S1.

**Enzyme**	**V_{max} (µmol·min^{-1}·mg^{-1})**	**K_M (mM)**	**k_{cat} (s^{-1})**	**k_{cat}/ K_M (M^{-1}·s^{-1})**
wtTPI	5044.4 ± 144.3	0.77 ± 0.07	4484 ± 128	(5.82 ± 0.56) x 10^6
pTPI	4639.6 ± 158.1	1.13 ± 0.13	4125 ± 141	(3.65 ± 0.44) x 10^6

Michaelis–Menten kinetic parameters of recombinant wtTPI and the pTPI were determined using GAP as substrate under standard assay conditions. Data are expressed as mean ± SEM derived from triplicate determinations.

Supplementary Table S2. List of the five highest-ranked binding poses for each PPI–TPI combination.

wtTPI			dTPI		pTPI	
**PPI**	**Vina score (kcal/mol)**	**Binding site**	**Vina score (kcal/mol)**	**Binding site**	**Vina score (kcal/mol)**	**Binding site**
Omz	−6.6	Dimer interface	−7.7	Dimer interface	−7.1	Dimer interface
Omz	−6.2	Other cavity	−7.5	Other cavity	−6.7	Other cavity
Omz	−5.9	Other cavity	−6.4	Other cavity	−6.6	Other cavity
Omz	−5.6	Other cavity	−5.7	Other cavity	−6.6	Other cavity
Omz	−5.6	Other cavity	−5.4	Other cavity	−6.1	Other cavity
Esz	−6.6	Dimer interface	−7.9	Dimer interface	−7.2	Dimer interface
Esz	−5.9	Other cavity	−7.2	Other cavity	−6.3	Other cavity
Esz	−5.9	Other cavity	−6.7	Other cavity	−6.3	Other cavity
Esz	−5.9	Other cavity	−5.9	Other cavity	−6.3	Other cavity
Esz	−5.8	Other cavity	−5.8	Other cavity	−6.1	Other cavity
Ptz	−6.4	Catalytic pocket	−7.7	Dimer interface	−6.7	Dimer interface
Ptz	−6.4	Other cavity	−7.6	Other cavity	−6.4	Other cavity
Ptz	−6.3	Dimer interface	−6.8	Other cavity	−6.2	Other cavity
Ptz	−5.9	Other cavity	−6.1	Other cavity	−6.2	Other cavity
Ptz	−5.4	Other cavity	−5.4	Other cavity	−6.2	Other cavity
Lsz	−6.6	Dimer interface	−8.0	Dimer interface	−7.1	Dimer interface
Lsz	−6.1	Other cavity	−7.5	Other cavity	−6.6	Other cavity
Lsz	−6.0	Other cavity	−6.8	Other cavity	−6.5	Other cavity
Lsz	−5.9	Other cavity	−5.9	Other cavity	−6.2	Other cavity
Lsz	−5.8	Other cavity	−5.3	Other cavity	−6.2	Other cavity
Rbz	−6.2	Catalytic pocket	−7.5	Dimer interface	−6.7	Dimer interface

Rbz	−5.8	Other cavity	−6.8	Other cavity	−6.2	Other cavity
Rbz	−5.6	Other cavity	−6.5	Other cavity	−5.8	Other cavity
Rbz	−5.6	Other cavity	−6.3	Other cavity	−5.7	Other cavity
Rbz	−5.5	Other cavity	−5.0	Other cavity	−5.5	Other cavity

Supplementary Table S3.

**Jurkat cells**	V_{max} **(µmol·min⁻¹·mg⁻¹)**	K_M **(mM)**
Control (without treatment)	9.98 ± 0.29	0.57 ± 0.58
+ Rbz (6 mM)	5.4 ± 0.098	0.76 ± 0.043

Michaelis–Menten kinetic parameters of endogenous TPI activity in Jurkat cell lysates were determined under control conditions (untreated) or following treatment with Rbz. Values for V_{max} and K_M were obtained using GAP as substrate under standard assay conditions. Data are expressed as mean ± SEM derived from triplicate determinations.

Supplementary Table S4. Comparative analysis of apoptosis percentages in normal T lymphocytes and Jurkat cells under different treatment conditions.

**Normal T lymphocytes**	**Viability (%)**	**Early apoptosis (%)**	**Late apoptosis (%)**	**Necrosis (%)**
Control	99.85	0.00	0.00	0.15
12 mM DCA	97.93	0.44	0.72	0.91
250 µM Rbz	92.59	3.11	2.18	2.12
500 µM Rbz	90.42	3.71	3.09	2.78
12 mM DCA + 250 µM Rbz	87.29	4.04	4.15	3.42
12 mM DCA + 500 µM Rbz	84.83	5.02	5.84	4.31
**Jurkat cells**	**Viability (%)**	**Early apoptosis (%)**	**Late apoptosis (%)**	**Necrosis (%)**
Control	99.59	0.00	0.00	0.41
12 mM DCA	96.99	0.25	0.95	1.81
250 µM Rbz	84.62	5.15	4.57	5.66
500 µM Rbz	55.87	9.36	24.64	10.13
12 mM DCA + 250 µM Rbz	42.26	10.84	33.13	13.77
12 mM DCA + 500 µM Rbz	17.11	9.87	45.23	27.79
